# Deep subsurface organic-rich shale supports abundant, diverse, and novel fungi

**DOI:** 10.1093/ismejo/wrag184

**Published:** 2026-07-29

**Authors:** Quinn S Moon, Elliott P Barnhart, Matthew S Varonka, Elizabeth J Tomaszewski, Michelle Orozco-Quime, Thomas Desrosiers, Ivan Paciorka, Michael Carley, James Schramski, Bradley S Stevenson, Magdalena R Osburn, Anurup Mohanty, Anna M Martini, Jason E Stajich, Jennifer C McIntosh, Timothy Y James

**Affiliations:** Department of Ecology and Evolutionary Biology, University of Michigan, Ann Arbor, MI 48108, United States; Institute for Global Change Biology, University of Michigan, Ann Arbor, MI 48108, United States; US Geological Survey, Wyoming-Montana Water Science Center, Helena, MT 59601, United States; US Geological Survey, Geology, Energy and Minerals Science Center, Reston, VA 20191, United States; US Geological Survey, Geology, Energy and Minerals Science Center, Reston, VA 20191, United States; Department of Ecology and Evolutionary Biology, University of Michigan, Ann Arbor, MI 48108, United States; Department of Ecology and Evolutionary Biology, University of Michigan, Ann Arbor, MI 48108, United States; Department of Ecology and Evolutionary Biology, University of Michigan, Ann Arbor, MI 48108, United States; Riverside Energy Michigan, LLC, Traverse City, MI 49684, United States; Riverside Energy Michigan, LLC, Traverse City, MI 49684, United States; Department of Earth, Environmental, and Planetary Sciences, Northwestern University, Evanston, IL 60208, United States; Department of Earth, Environmental, and Planetary Sciences, Northwestern University, Evanston, IL 60208, United States; Department of Earth, Environmental, and Planetary Sciences, Northwestern University, Evanston, IL 60208, United States; Geology Department, Amherst College, Amherst, MA 01002, United States; Department of Microbiology and Plant Pathology and Institute for Integrative Genome Biology, University of California Riverside, Riverside, CA 92521, United States; Department of Hydrology and Atmospheric Sciences, University of Arizona, Tucson, AZ 85721, United States; Department of Ecology and Evolutionary Biology, University of Michigan, Ann Arbor, MI 48108, United States; University of Michigan Herbarium, Ann Arbor, MI 48108, United States

**Keywords:** fungi, deep subsurface, carbon and energy fluxes, methanogenesis, shale

## Abstract

As Earth’s principal reservoir of organic carbon and microbial biomass, the deep subsurface hosts microorganisms capable of mobilizing this once-sequestered carbon. Contrary to standard assumptions of eukaryotic scarcity, this study documents abundant fungal communities, ranging from 4.2 × 10^3^ to 6.8 × 10^3^ fungal cells ml^−1^, across a methane-producing organic-rich shale 247–556 meters below the surface. Although fungal:bacterial cell ratios ranged from 1:7028 to 1:713, application of biomass conversion factors developed for oceanic systems yielded a median fungal:bacterial biomass ratio of 1:4.7. 16S ribosomal ribonucleic acid (rRNA) gene amplicons revealed bacterial and archaeal communities mirroring those found in well-characterized extremophilic, carbon-degrading environments, while sequencing of 18S rRNA gene and internal transcribed spacer rRNA spacer amplicons collectively identified a eukaryotic hotspot with 689 fungal operational taxonomic units across six phyla. The dominant fungal classes, Agaricomycetes and Dothideomycetes, are well-established degraders of recalcitrant carbon compounds at the surface, suggesting they may similarly contribute to organic matter degradation and ecosystem maintenance in the subsurface. Cultivation and isolation efforts yielded 205 fungal strains, including 13 candidate novel taxa, underscoring the deep subsurface as an underexplored eukaryotic habitat. Stable carbon isotopes indicate methane is predominantly generated via microbial conversion of the fossil carbon, while water isotopes suggest *in situ* geochemical conditions have been relatively stable since the Late Pleistocene, with subglacial recharge as a plausible mechanism for microbial introduction. Collectively, these findings suggest that fungi are underrecognized contributors to organic matter transformation and functional diversity in the deep biosphere, revealing a critical gap in our understanding of deep subsurface ecosystem processes.

## Introduction

The deep subsurface biosphere contains the majority of the planet’s microbial cells [1] and although regions of the deep subsurface are oligotrophic, ~90% of the planet’s organic carbon is also sequestered there [2]. Many oil, shale, and coal deposits support active microbial ecosystems, with buried sedimentary carbon persisting for use as electron donors and carbon sources, thereby enabling sustained activity and temporal persistence [3]. Under favorable geochemical conditions, microbial processes can deplete substantial portions of subsurface carbon reservoirs, such as ancient shallow oil fields [4], releasing much of their stored carbon into the atmosphere as methane (CH₄) and carbon dioxide (CO₂) [5]. Long believed to exclusively contain bacteria and archaea, a growing number of studies have detected eukaryotes, especially fungi, across the subsurface [6, 7]. Yet, the presence, composition, and functional roles of eukaryotes within hydrocarbon-degrading subsurface ecosystems remains unclear and are seldom integrated into biogeochemical models. Investigation is particularly pressing, as these reservoirs may serve as biodiversity hotspots by circumventing the carbon and energy limitations common in the oligotrophic subsurface and, when inoculated, can alter the global cycle and long-term storage of organic carbon in the Earth’s crust [8, 9].

Microbial gasification of subsurface carbon reservoirs constitutes a substantial, and potentially dominant, source of methane within Earth’s crust [10]. Historically, such methanogenesis was thought to result from the synergistic interactions between degradative bacteria and methanogenic archaea, with bacteria driving the hydrolysis of recalcitrant hydrocarbons; however, increased detection of fungal taxa now challenges this view [6, 11–13]. On the surface, highly recalcitrant carbon sources, particularly those containing long-chain and aromatic substrates such as oil, shale, and coal, are more efficiently degraded by fungi than by bacteria [14]. Specifically, fungi in the Basidiomycete order Polyporales, and many Ascomycete fungi are well documented to readily decompose both shale and coal, including under anoxic conditions [12, 15–17]. Although the mechanisms underlying fungal decomposition in anoxic environments remain incompletely characterized, under aerobic conditions, saprobic fungi demonstrate a pronounced competitive advantage due to their ability to form penetrative hyphal networks and their extensive repertoire of carbohydrate-active enzymes [18].

Bacterial and archaeal densities in the deep subsurface are generally comparable to those observed in freshwater and marine environments, typically ranging from 10^4^ to 10^6^ cells/ml of water [19, 20]. Although microbial densities generally decrease with increasing depth below the surface [20], cell abundances in carbon-rich subsurface layers can increase significantly, with concentrations reported as high as 10^7^ cells/ml of groundwater [21]. Estimations of subsurface microbial biomass have generally been based on deoxyribonucleic acid (DNA) staining techniques that presume all detected cells are either bacterial or archaeal, which likely underrepresents eukaryotic populations [19–21]. Without previous quantification, the presence of fungi in subsurface environments is typically considered negligible or transient [1, 4, 20, 22, 23]. The omission of eukaryotes from such assessments has largely stemmed from the prevailing view that eukaryotic anaerobes are uncommon, compounded by the incomplete understanding of the distribution of anaerobic metabolic pathways across eukaryotic taxa. Nonetheless, emerging evidence increasingly indicates that many Dikarya and non-Dikarya fungal lineages possess metabolic capabilities of facultative anaerobes [12, 18, 24–28], suggesting a greater ecological versatility in anoxic settings than previously recognized. Widespread detection of fungal communities has further demonstrated that fungi fulfill critical ecological roles in other aquatic and extreme environments previously believed to be inhospitable, such as hydrothermal vents, hot springs, and hypersaline lakes [29–31].

This study investigates the role of fungi in remineralizing carbon within the organic-rich, fractured, and low-maturity Antrim Shale of the Michigan Basin, which formed in the Upper Devonian (382.7–358.9 Ma). The buried shale is the one of the largest sources of biogenic methane in the global subsurface [32]. Although bacterial and archaeal communities here are documented to contain heterotrophic bacteria and methanogenic archaea [8, 33], it remains unknown whether eukaryotes inhabit this ecosystem that has likely been recharged with water and generated microbial gas several times over [33]. The abundance of recalcitrant, yet seemingly bioavailable, carbon in the Antrim Shale, coupled with a well-defined geological and hydrological setting, offers an ideal context for studying microbial dynamics representative of sedimentary environments worldwide. We used an innovative approach consisting of combined amplicon sequencing targeting fungi, eukaryotes, bacteria, and archaea with two methods of total and fungal biomass estimation in parallel with stable isotope and geochemical analyses of the sampled formation waters. We sought to assess fungal diversity within the broader microbiome, hypothesizing that while fungal biomass and cell abundance would be lower than those of bacteria, fungi would nonetheless represent a substantial component of subsurface biomass. In addition, a culture-based approach targeted the isolation of dominant fungal taxa, anticipating that some would be cultivable and that these isolates might represent putative novel species. Collectively, this study demonstrates that fungi comprise a considerable portion of subsurface biomass in the Antrim Shale, indicating their potential role in biogeochemical cycling and emphasizing the benefits of including deep subsurface environments in global biodiversity assessments.

## Materials and methods

### Site description and sampling

**Figure 1:**
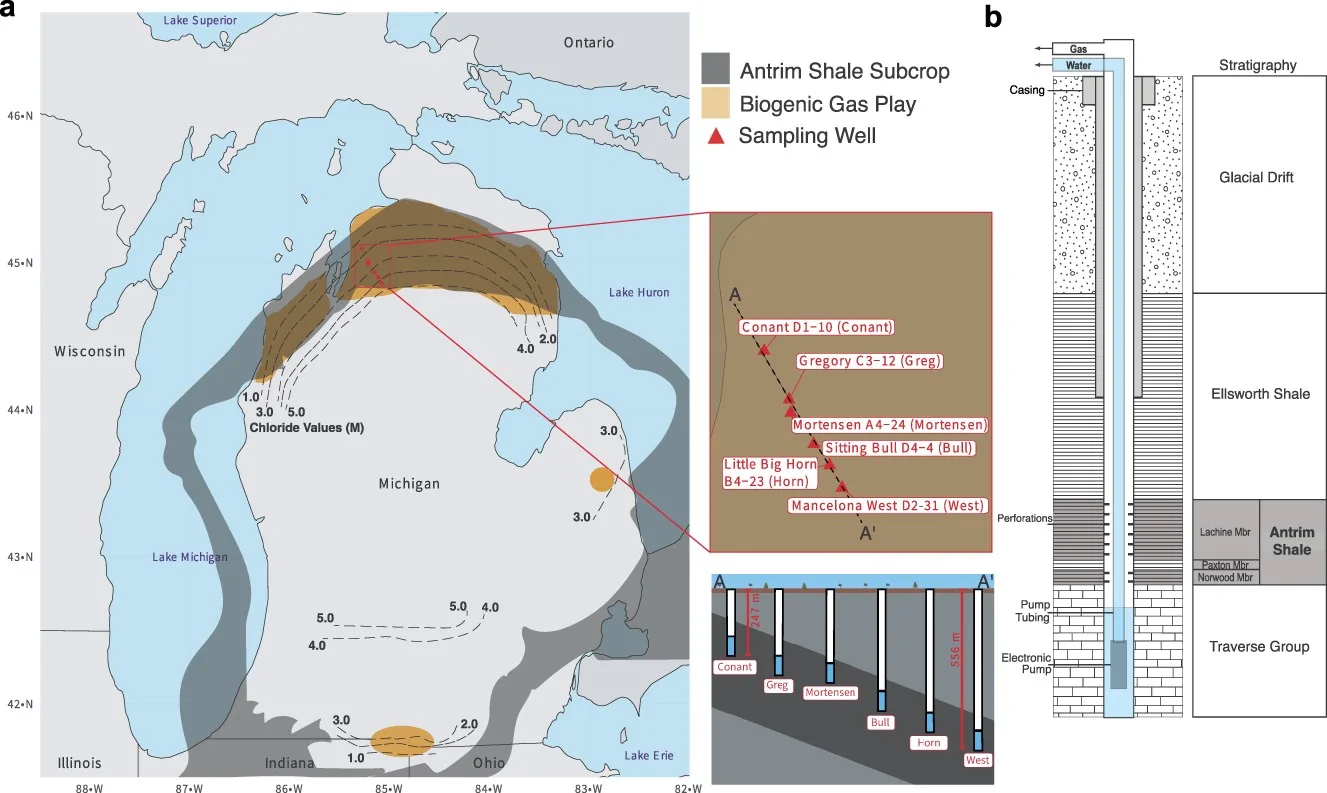
Sampling diagram of formation waters within the buried Antrim Shale. (a) Map and schematic diagram illustrating the locations of sampling wells within the Antrim Basin, Michigan, USA. The distribution of shale and gas plays is depicted, and the extensively characterized chloride (Cl^−^) gradient is indicated by dashed lines (adapted from [34]). Well locations are indicated with triangles, and the relative depths are displayed. (b) Illustrated alongside regional stratigraphic layers, a schematic representation of a formation water well equipped with an electronic pump and tubing assembly installed within the Antrim Shale.

Gas and co-produced formation water samples were collected directly at the wellhead from actively producing shale-gas wells in Antrim County, Michigan (Fig. 1). Due to extensive hydrocarbon exploration in the Michigan Basin, the hydrostratigraphic architecture, fluid chemistries, and rock properties of the Antrim Shale are well understood [32, 35]. The selected wells were drilled to depths ranging from 247 to 556 meters below the surface to access the buried Antrim Shale formation, with perforations made in the well tubing at the depth corresponding to the shale interval, enabling formation water to enter and flow to the pump positioned at the bottom of each well (Fig. 1). Eighty liters of formation water was collected directly from each well into sterile containers. For DNA analysis, samples were filtered in triplicate using a Masterflex Peristaltic Pump (Masterflex, Gelsenkirchen, Germany) employing 0.22 μm Millipore polycarbonate filters (EMD Millipore, Billerica, MA). Filtration continued until either 20 liters of sample was processed or the filters became clogged. The 0.22 μm filters were immediately flash frozen on dry ice and stored at −80°C until further processing. For fungal culturing, 1.2 μm polycarbonate filters (EMD Millipore, Billerica, MA) were instead used, and samples were filtered as described above. For abundance quantification, water was fixed with glutaraldehyde to a final concentration of 2% before flash freezing on dry ice and stored at −80°C until further processing. Gas samples were collected in IsoTube cylinders (Isotech, Champaign, IL) connected to the wellhead and flushed ten times with produced gas before filling.

### Geochemical analyses of the formation waters

Aqueous geochemistry, water isotope, and gas isotope methods and data are publicly available through a US Geological Survey (USGS) data release [36] (Supplementary Table 1). In short, temperature, pH, and specific conductance of formation waters were immediately measured in the field. Samples were analyzed for a suite of bulk geochemical parameters including total dissolved solids (TDS), non-purgeable dissolved organic carbon (NPDOC), dissolved inorganic carbon (DIC), alkalinity, total dissolved nitrogen (TDN), and major ions at the USGS research center in Reston, Virginia. All sampled wells have been in operation and pumping water daily for at least 10 years since initial drilling, or last workover, and each has produced a cumulative total exceeding 1 million liters of water during this period.

### Constraining water inundation history and gas origin

Water stable isotopes were analyzed by continuous flow-isotope ratio mass spectrometry (CF-IRMS) at Isotech (Champaign, IL) and off-axis integrated cavity output spectroscopy (OA-ICOS) in cooperation with University of Massachusetts and Smith College. Gas compositional data were obtained through gas chromatography, and gas-phase d^2^H and δ^13^C of CH_4_ (C_1_) were measured via isotope ratio mass spectrometry at Isotech. Methane isotopologues were also analyzed using a tunable infrared laser direct absorption spectrometer at Massachusetts Institute of Technology, using resulting data to estimate the apparent formation temperature [37].

### Deoxyribonucleic acid extractions, library preparation, and sequencing

DNA isolation from frozen filters was carried out as previously described [38]. In short, phosphate-buffered saline solution was used to release biomass from the filters and DNA was extracted from the resulting pellets using the QIAGEN PowerWater Kit (MoBio Laboratories, Inc., Carlsbad, CA). Negative DNA extraction controls were quantified using a Qubit fluorometer at maximum volume, yielding undetectable DNA concentrations, and were run on a 0.5% agarose gel to confirm the absence of contamination; these controls were also subjected to internal transcribed spacer (ITS) amplification using ITS1/ITS4 [39], and showed no visible bands on a 1% agarose gel. Extracted DNA was submitted to the Integrated Microbiome Core (IMR) at Dalhousie University for gene amplification, library preparation, and paired end 2 × 300 bp MiSeq System (Illumina) sequencing of the fungal ITS2 region, the general eukaryotic 18S rRNA gene, and bacterial and archaeal 16S rRNA gene (see Supplementary Information).

### Cell abundance and biomass estimates

Microbial cell abundance in the sampled waters was assessed using epifluorescence microscopy. In brief, the glutaraldehyde fixed cells were vacuum filtered onto black polycarbonate filters (0.2 μm pore size). For total microbial cell counts, the filters were stained with 4′,6-diamidino-2-phenylindole (DAPI) and fungal counts were enumerated by staining with Calcofluor White (CFW). For DAPI stained samples, 300 cells were counted across a minimum of 20 randomly selected fields of view (FOVs) and CFW stained samples were quantified across 30 FOVs. Cell counts were used to calculate microbial and fungal abundance per unit volume of the original water sample.

The relative abundance of fungi to bacteria was determined using the BactQuant TaqMan 16S rRNA gene assay [40] and the FungiQuant TaqMan 18S rRNA gene assay [41], employing previously described primers and probes, on a QuantStudio™3 real-time polymerase chain reaction (PCR) system (Thermo Fisher Scientific, Waltham, MA). For each sample, three independent quantitative PCR (qPCR) assay were performed for each gene and abundance estimates were made relative to dsDNA oligonucleotide standards (gBlocks) and corrected to genome counts. Cell ratios were converted to biomass ratios using the conversion factor of 10 fg C bacterial cell^−1^ and 6469 fg C fungal cell^−1^ [42, 43] (see Supplementary Information; Supplementary Fig. 1).

### Amplicon sequence processing and analysis

Initial processing and quality control of demultiplexed raw rRNA gene amplicon data sequence data for all three primers sets (ITS2, 16S, 18S) were performed using QIIME 2 (v2021.11 81) [44] with DADA2 [45] plugin for denoising and chimeric sequence removal. 16S rRNA gene sequences were retained as amplicon sequence variants (ASVs) and ITS and 18S rRNA gene sequences were clustered at 98% and 99%, respectively. 16S sequences were classified with the SILVA database [46], 18S sequences with PR2 [47] for microeukaryotes and SILVA for fungi [46], and ITS2 sequences were classified using a BLASTn-based approach against the UNITE database [48], applying taxon-specific e-value and similarity thresholds at the level of species to phyla [49, 50]. For ITS2 fungal analyses, all non-fungal sequences were excluded (5.4%). In the 18S dataset, operational taxonomic units (OTUs) classified as land plants (division Streptophyta), vertebrates (class Craniata), or arthropods (class Arthropoda) were dropped from the analysis and accounted for 64.5%, 0.26%, and <0.01% of reads and 49, 1, and 1 OTUs, respectively. Based on saturation curves and commonly applied minimum read depth thresholds [50], samples containing fewer than 320 quality trimmed 18S rRNA gene reads or fewer than 300 quality trimmed ITS reads were excluded from downstream analyses, whereas all 16S rRNA gene samples were retained (Supplementary Figs 2 and 3). Replicates from the same well exhibited strong clustering in non-metric multidimensional scaling analyses and were therefore pooled for downstream analyses (see Supplementary Information; Supplementary Fig. 4).

### Fungal isolation and identification

After filtering formation water, the 1.2 μm filters were placed in sterile ddH_2_O and manually shaken vigorously to dislodge attached cells. A 500 μL aliquot of the resulting suspension was spread in five replicates onto each of two types of solid agar media: PDA and GY5. Plates were incubated at 17°C to approximate natural temperatures. Emerging fungal colonies were isolated into axenic culture. DNA was extracted from all axenic fungal cultures using a CTAB protocol [51], the ITS region was amplified with ITS1/ITS4, [39], and amplicons were Sanger sequenced using ITS1. To compare isolate ITS sequences with ITS community amplicon sequencing data, the ITS isolate sequences were used to create a custom database for BLASTn searches against the amplicon OTUs. Isolates were classified as a match if their sequences exhibited ≥98% similarity to an amplicon sequence, as determined by pairwise alignment. Isolates meeting this criterion were clustered accordingly and assigned the taxonomic designation of the corresponding amplicon sequence. Isolate ITS sequences not present in the ITS amplicon dataset were clustered into 98% OTUs and classified using the pipeline described above (see Supplementary Information).

### Phylogenetic analysis

The isolate OTU with the lowest sequence identity to any UNITE reference (74%) was further characterized using phylogenetic inference. The previously generated DNA extract was used to amplify and Sanger sequence the 28S large ribosomal subunit (nrLSU) using the primer pair LR0R/LR7 [53]. Forward and reverse Sanger sequences were trimmed and merged to form a consensus sequence using Geneious v10.1.2 [54]. BLAST searches and reviewing of relevant literature was used to build the reference LSU dataset. LSU sequences were aligned using MAFFT [55] with default parameters, alignment trimmed with trimAL [56], and a max likelihood tree was produced in IQ Tree with 100 bootstrap replicates [57].

### Additional contamination controls

Controls were included in each step to avoid and monitor contamination. DNA extractions and culturing work was completed in a laminar flow hood. In addition, sterile water was spread onto both media types as a negative control. Both media types were also exposed to ambient air for 5 minutes to assess potential airborne contamination. All colonies arising on control plates were isolated and sequenced using the same protocols as for experimental samples. In total, 23 control isolates were obtained, representing 12 distinct culture OTUs; eight of these 12 matched an amplicon OTU from the community dataset at >98% ITS sequence similarity. Isolates and amplicon OTUs detected in isolation controls were excluded from downstream analyses.

### Statistical analysis

All statistical analyses and data visualizations were performed in R v4.3.1 [58]. To evaluate relationships between fungal diversity, total microbial diversity, and environmental conditions, we focused on a limited set of variables, including diversity indices (observed richness, Shannon diversity, Hurlbert’s PIE evenness), abundance, and key geochemical parameters (pH, NPDOC, DIC, TDN, salinity, and depth). Relationships among variables were evaluated using linear models and Spearman’s rank correlations implemented in R using the default *stats* package [58]. Rather than relying solely on statistical significance, we interpreted the magnitude and direction of effect sizes to assess ecological relevance (see Supplementary Information).

## Results and discussion

### Gas and water origins

**Figure 2:**
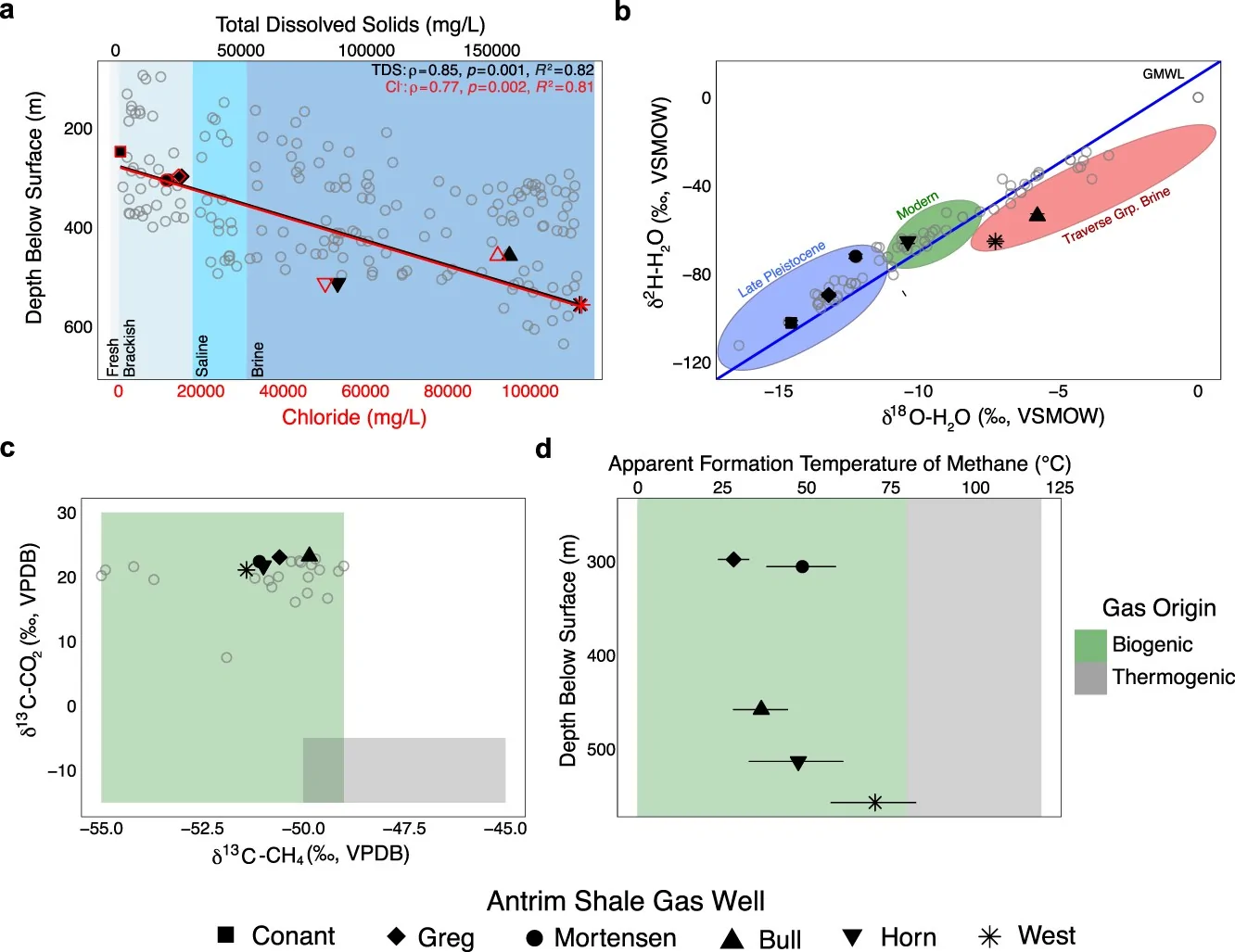
Gas and co-produced formation waters across the Antrim Shale. (a and b) Formation water results from sampled Antrim Shale wells: (a) TDS and chloride concentrations versus well depth with their Spearman’s rank correlation coefficient (ρ) and *P*-value; the R^2^ value from a linear regression fit is also reported. (b) Stable water isotope composition, measured with the Vienna Standard Mean Ocean Water (VSMOW) standard, displayed with the GMWL [62], late Pleistocene subglacial meltwater and modern precipitation [63], and underlying Traverse Group basinal brine [64]. (c and d) Results of the produced gasses are shown with shaded regions indicating established values for microbial versus thermogenic origin [65–67]. (c) Carbon isotopic ratios of produced CO_2_ and CH_4_ relative to the Vienna Pee Dee Belemnite (VPDB) standard. (d) Apparent formation temperature of methane based on measured isotopologue distributions. Black symbols represent new formation water data from this study, compared to previously published values for the Antrim Shale (open gray circles) [8, 34, 35, 68, 69] (Supplementary Data 2). Where available, error bars indicate ±1 standard error (SE). See Supplementary Fig. 9 and Table 1 for complete geochemical results.

Previous research suggests that subsurface microbial populations are strongly modulated by both their interaction with surface systems and prevailing geochemical conditions [59, 60]; therefore, analyses focusing on subsurface microbiology must be contextualized within the broader hydrological system [61]. In the Michigan Basin, multiple advances and retreats of continental ice sheets over the past ~2 million years dilated natural fractures and enhanced water recharge into the Antrim Shale [35]. Accordingly, sampled formation waters exhibited a pronounced salinity gradient with depth and distance from the shale subcrop, with chloride concentrations rising from fresh water in the Conant well (448 mg/L TDS at 247 m depth) to highly saline conditions in Mancelona West (West) well (112 000 mg/L TDS at 556 m depth) (Fig. 2a). The observed salinity gradient is characteristic of many other sedimentary basins where remnant brines, formed by evaporation of ancient seawater and/or dissolution of salt deposits, are retained at depth and have been diluted by paleo- to modern-meteoric recharge along basin margins [70, 71]. Moreover, dissolved oxygen concentration is a critical determinant of microbial community composition, but is difficult to measure accurately *in situ*, as pumping water to the surface can introduce trace amounts like those measured in our sites (0.1%–0.6%; Supplementary Table 1). Although widespread archaeal methanogenesis, as described previously for the Antrim Shale [8, 33], indicates prevailing anoxic conditions, the possibility of endogenous oxygen production, such as dark oxygen via microbial dismutation, has recently been proposed as an additional source in other ancient subsurface waters [21].

The isotopic composition of Antrim Shale formation waters (Fig. 2b) ranged from relatively low *δ*^18^O-H_2_O and *δ*^2^H-H_2_O values for fresh to brackish waters, consistent with late Pleistocene glacial meltwater (Mortensen, Greg, and Conant wells, shown in blue), to relatively high values for the most saline samples that plot to the right and below the Global Meteoric Water Line (Sitting Bull (Bull) and West wells, shown in red) (GMWL; [62]), indicative of remnant basinal brines (Fig. 2b; [8, 63, 71]). Brine from the Little Big Horn (Horn) well plots within the field of local modern precipitation and has a slightly lower salinity than expected (below trendline in Fig. 2a, shown in green), which likely indicates some component of more recent recharge through the underlying Traverse Group, a limestone with higher hydraulic conductivity. Collectively, these analyses suggest that the current favorable geochemical conditions in the shale have been relatively stable since the Late Pleistocene and suggest subglacial meltwater recharge as a plausible mechanism and timeline for microbial introduction from near-surface environments [5].

Dissolved methane concentrations in the formation waters remained consistently elevated across the transect, comprising 86% to 97% of the total gas content (Supplementary Fig. 5). While deep subsurface methane can be formed through thermogenic processes, and secondary microbial activity can obscure signals of microbially produced gasses, the high DIC values (ranging between 6.9 and 23.6 mmol/L; Supplementary Table 1) combined with high *δ*^13^C-CO_2_ values (>20‰; Fig. 2c) indicate ongoing microbial activity in the formation waters [8, 68]. Both the *δ*^13^C-CO_2_ and *δ*^13^C-CH_4_ values (Fig. 2c) and predicted formation temperature of methane (Fig. 2d), suggest that most of the methane is of microbial origin, derived from carbon previously stored in the shale deposit. The organic-rich Antrim Shale contains up to 25 wt% organic matter, primarily composed of bitumen and preserved assemblages of planktonic algae and wood [72]. Moreover, erosion linked to late Pleistocene glacial retreat caused the liberation of as much as 88% of the methane previously stored within the shale matrix [5], underscoring the significant migration potential of methane out of subsurface systems over time. Microbial colonization and the subsequent transformation of organic carbon occur under similar burial conditions in sedimentary basins worldwide [73]. Therefore, microbial transformations within organic-rich strata may have a direct, though not yet completely characterized, impact on global greenhouse gas concentrations [5, 74, 75].

### Fungi are common and constitute a fifth of the biomass in the Antrim Shale

**Figure 3:**
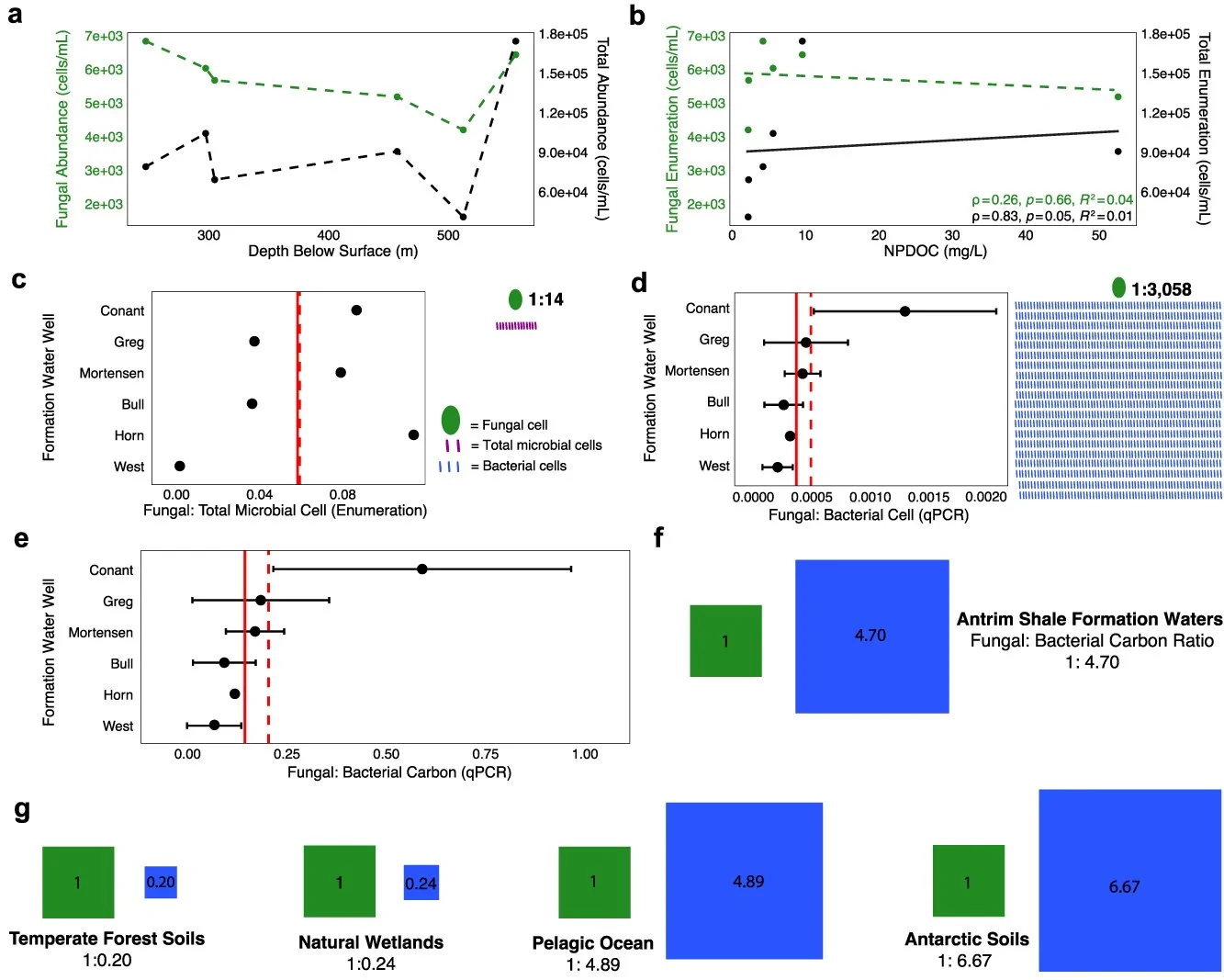
Total and fungal cell abundance based on enumeration microscopy. (a) Cell abundance in sampled formation waters, based on enumeration of fungal cells (CFW) and total cells (DAPI), plotted against depth (see Supplementary Fig. 4 for representative fields of view). (b) Green dashed and black solid lines represent linear regression fits (least-squares method) between NPDOC and fungal or total cell abundance, respectively; total abundance values are scaled to align with the fungal axis, and a solid trend line denotes a statistically significant relationship. (c) Ratio of fungal to total cells, based on enumeration. (d) Ratio of fungal to bacterial cells based on 18S and 16S qPCR, respectively; with each point representing the mean of technical replicates (error bars show ±1 SE; no error bar for single replicates). (c and d) Vertical red lines indicate the median across all samples, and dashed red lines indicate the mean. Median cell ratios are graphically displayed with fungi (green), bacteria (blue), and total microbial cells (purple). (f) Median biomass ratios are graphically displayed for fungi (green) and bacteria (blue). (g) Previously reported fungal: Bacterial biomass ratios in natural wetlands and temperate forests [76], the pelagic ocean [42], and Antarctic soils [77].

We used epifluorescence microscopy to quantify microbial biomass along the pronounced depth and salinity gradients of the northern Michigan Basin. The total cell abundance within the sampled formation waters, determined by DAPI, ranged from 4.1 × 10^4^ to 6.9×10^4^ cells/ml (Fig. 3a; Supplementary Data 1). Fungal cell abundance, assessed via CFW, ranged from 4.2 × 10^3^ to 6.8 × 10^3^ fungal cells/ml (Fig. 3a). Although we were unable to locate previous estimates of fungal biomass in subsurface waters, the values observed here are comparable to, or perhaps slightly higher than, those reported in marine environments, which range from 8.1 × 10^0^ to 1.98 × 10^3^ fungal cells/ml [42, 78]. Additionally, over 99% of the observed fungal biomass consisted of single cells. This pattern again aligns with findings from aquatic environments, where yeasts and dimorphic groups typically dominate open water habitats [42, 79]. Using established conversion factors for fungi (6469 fg C per fungal cell) [42], which were derived from pelagic communities dominated by Dikarya and yeast forms, our calculated CFW based fungal biomass ranged from 27.17 to 44.25 μg C/L. These values fall between ergosterol based fungal biomass ranges reported in the pelagic ocean (0–3.03 μg C/L) [42], arctic coastal waters (0.01–13.35 μg C/L) [80], and coastal waters highly impacted by anthropogenic activities (110–280 μg C/L) [81]. Comparison between methods and subsequent interpretation must account for differences in specificity, including the absence of both chitin and ergosterol from several zoosporic lineages, the presence of chitin in various protistan and animal groups [52, 82, 83], and the affinity of CFW for both chitin and cellulose, which may result in non-specific labeling [84]. Such factors can cause CFW-based estimates to be up to twice as high as those derived from ergosterol-based methods [42]. Nonetheless, estimates of fungal abundance and biomass obtained via CFW staining indicate that fungi are a consistent component of the Antrim Shale microbiome, with values broadly comparable to those reported for marine ecosystems.

Group specific qPCR was employed to determine the relative abundance of fungal and bacterial biomass. Across all sites, the fungal:bacterial cell ratio ranged from 1:7028 (SE 1:6669) to 1:713 (SE 1:1107), with a median of 1:2572 (Fig. 3d). After adjusting cell count ratios using biomass conversion factors [42], the calculated ratio of fungal to bacterial carbon spanned from 1:10.86 (SE 1:10.31) to 1:1.10 (SE 1:71), with a median of 1:4.7 (fungal carbon: bacterial carbon)(Fig. 3d). This median value suggests that fungal carbon in the Antrim Shale makes up a far smaller proportion compared to ratios from natural wetlands and temperate forests [76] and are more similar to values reported from the pelagic ocean [42], and Antarctic soils [77] (Fig. 3g). However, subsurface specific biomass conversion factors for fungi are currently unavailable. Consequently, these comparisons rely on conversion factors developed for pelagic organisms, which have not yet been validated for subsurface fungal communities [42]. Biomass conversion factors are widely applied to cell count data from both terrestrial and marine environments [42, 80, 85, 86], with published marine estimates of fungal cellular carbon content ranging from 6469 to 203 750 fg C fungal cell^−1^ [42, 80]. The formation waters sampled here share several characteristics with saline aquatic environments, including broad similarities in fungal community composition, the predominance of unicellular fungal morphologies, dissolved organic carbon concentrations, salinity, and aquatic habitat structure. We therefore applied recently developed pelagic fungal conversion factors because they are based on combined measurements of cell biovolume, ergosterol content, and cell dry mass. In addition, the lower conversion factor applied here, 6469 fg C fungal cell^−1^, provides a relatively conservative approximation, which may be particularly relevant if subsurface fungal cells are smaller than their surface counterparts, as has been reported for bacteria [87]. Nevertheless, differences in inferred cell ratios and biomass among methodological approaches are well recognized, and estimates of fungal:bacterial dominance are especially sensitive to the methods used [88]; molecular approaches such as qPCR typically produce lower relative values for fungal abundance [14]. Our protocol relied on sampling free-living cells by filtering formation water, which could underestimate the overall microbial abundance as 20%–80% of microbial biomass in similar environments is attached to biofilms rather than suspended in the water column [1, 89, 90]. Therefore, these biomass estimates can be interpreted as first-order approximations rather than uniform subsurface values. Future investigations using subsurface incubations provide a promising approach to investigate the diversity and function of biofilm-associated microbial communities in these environments [91, 92].

Both molecular (qPCR) and microscopy-based analyses revealed a substantial presence of fungal biomass, with observed concentrations and ratios that parallel those in other environmental systems, particularly marine settings (Fig. 3f and g). The similarity in measured absolute and relative biomass to those in pelagic oceans may point to ecological parallels (Fig. 3g; [42]). Although fungal and total microbial cell counts were not strongly linked to sampling depth, total cell abundance did significantly correlate with concentrations of NPDOC (*R*  ^2^ = 0.012, *P* = .05; Fig. 3b). Although the correlation is weak, it may suggest that dissolved organic carbon (DOC) availability may directly influence the densities of free-living cells—and conversely, that active microbial communities facilitate the solubilization of DOC from shale deposits. The measured range of DOC (2.2–52.6 mg C/L; median 6.96 mg C/L) is similar to reported values from oceanic environments (6.12–16.2 mg C/L) [93–96], which may account for the comparable planktonic microbial abundances across systems. The detection of substantial fungal populations runs counter to the prevailing view that eukaryotes are absent or scarce in deep subsurface settings. As the fundamental roles of bacteria in subsurface environments are well documented, it becomes increasingly important to consider the ecological contributions of aquatic fungi as well.

### Diversity patterns across the depth and salinity gradient

**Figure 4:**
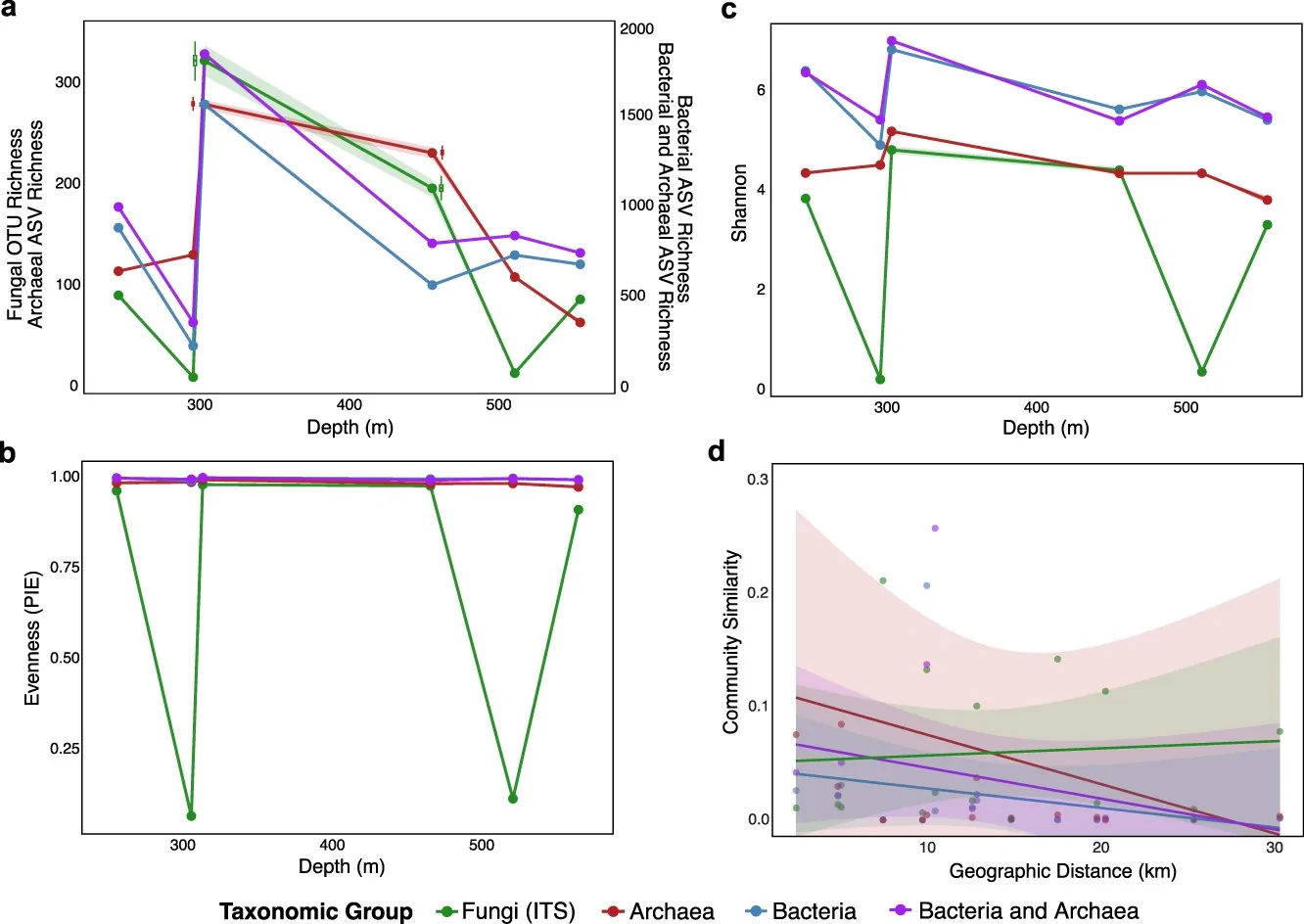
Multi-panel plot illustrating key diversity metrics across formation well sites for distinct taxonomic groups, each represented with separate colors. (a) Observed richness at each site, (b) Shannon diversity index, (c) community evenness, and (d) distance decay relationships for each taxonomic group; lines represent the fitted regression slopes, and shaded regions denote the SE for each slope estimate. (a–c) Alpha diversity estimates were calculated from 1000 repeated rarefactions where for each marker gene, samples were randomly subsampled to the minimum read depth observed for that group (36 249 for 16S and 1690 for ITS). Points and lines show the mean estimate across rarefactions, shaded regions show the empirical 95% interval, and box and whisker plots are displayed for points whose 95% interval exceeds the thickness of the point. The 18S rRNA gene dataset was not included because three sites did not meet the minimum read-depth threshold for analysis. The solid lines are shown solely for visual clarity and do not imply statistical modeling or significance. No significant relationships were detected between richness, evenness, or Shannon diversity and well depth for any group (*P* > .05; a–c). For complete taxonomic stacked bar plots see Supplementary Fig. 7.

Amplicon sequencing of formation waters from all sampled wells generated 75 537 reads representing 689 ITS OTUs, 65 890 reads representing 342 18S rRNA gene OTUs, and 1 136 789 reads representing 6099 16S rRNA gene ASVs. Three wells (Conant, Greg, and Horn) were excluded from subsequent 18S rRNA gene analyses because none of their technical replicates yielded sufficient sequencing depth (>320 reads). The 16S rRNA gene sequences were retained as ASVs, whereas ITS and 18S rRNA gene sequences were clustered into OTUs at 98% and 99% sequence identity, respectively, because of well documented intragenomic variation in fungi and microeukaryotes [50]. These commonly applied sequence processing approaches [50] are unlikely to substantially affect the overall results [97]. Rarefaction curves for each included well and primer set reached saturation and recovered at least 99% of Chao1 estimated richness (Supplementary Figs 2 and 3), indicating that sequencing depth was generally sufficient to characterize the sampled microbial communities. However, observed diversity increased substantially as additional wells were included (Supplementary Fig. 3), suggesting that the dataset captures only a portion of the total microbial diversity present within the Antrim Shale. Within sites, ITS read counts and fungal OTU richness were strongly correlated (*P* = .03, ρ = 0.89; Supplementary Fig. 6). Moreover, 16S ASV diversity and ITS fungal richness exhibited a significant positive correlation across wells (*P* = .03, ρ = 0.37), indicating that sites with higher bacterial and archaeal diversity also harbored increased fungal diversity (Supplementary Fig. 6). Microbial communities exhibited unique compositions in each well, with no single clade dominating across all sampled locations (Fig. 4d; Supplementary Fig. 7). Specifically, no 16S gene ASV was detected in every well, while only 4 out of 342 18S gene OTUs and two out of 749 ITS OTUs were universally present. The observed diversity and spatial segregation likely indicate low hydraulic connectivity and limited flow rates in these habitats, relative to surface systems [34].

### Fungal isolation

**Figure 5:**
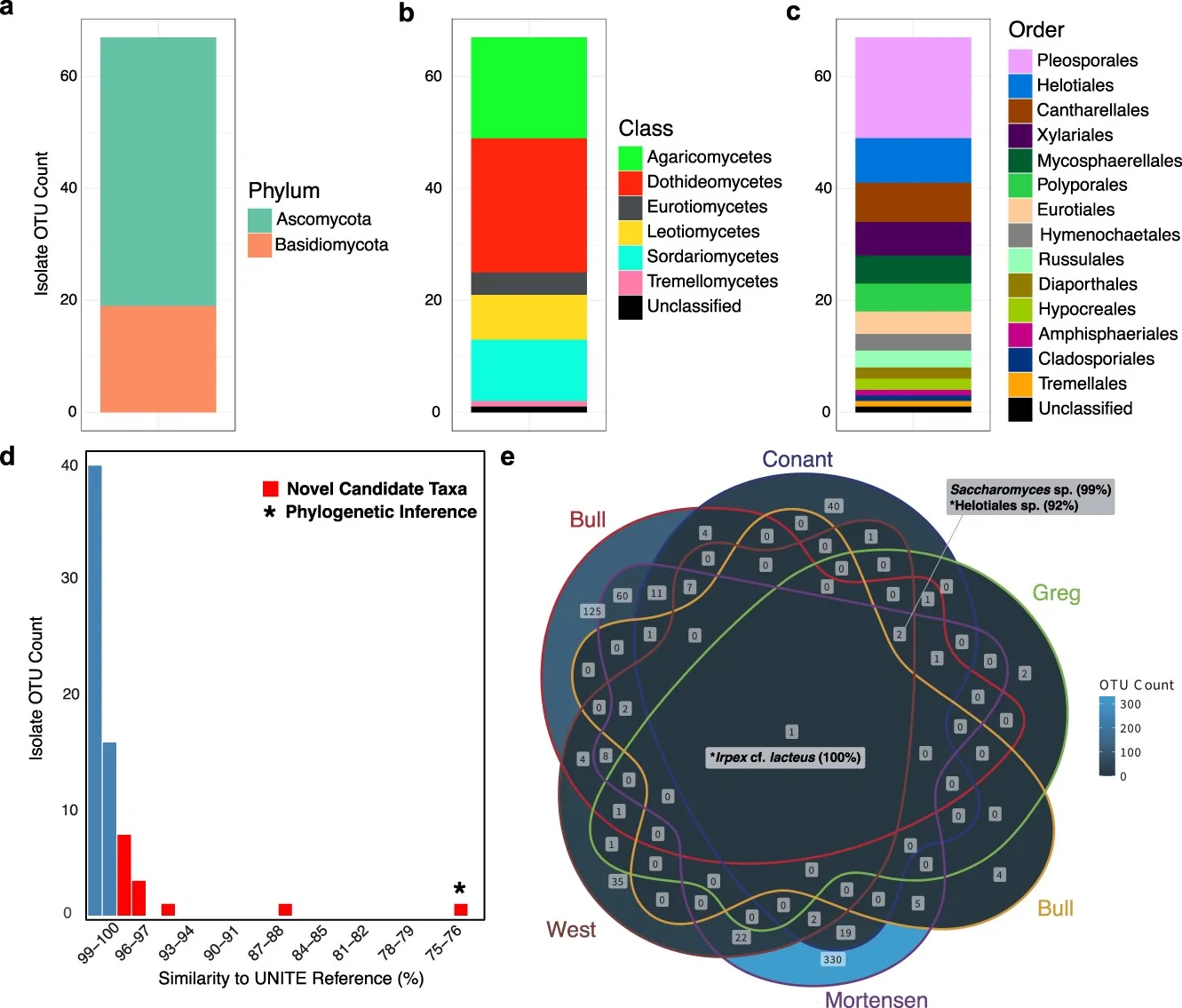
Multi-panel plot displaying characteristics of isolated fungi from methane producing wells. (a–c) Taxonomic stacked bar plots of isolates summarized at various taxonomic levels. (d) Distribution of sequence similarity between isolates and entries in the UNITE fungal database. (e) Number of amplicon ITS OTUs shared among sites, with the single OTU found at all sites and the two OTUs present at five out of six sites highlighted; an asterisk (*) denotes that a representative of that OTU was successfully cultured.

Fungal isolation from filtered formation water samples led to the establishment of 205 axenic cultures. Following ITS barcoding and OTU clustering at a 98% similarity threshold, 67 distinct culture-derived OTUs were identified, 34 of which matched (>98% similarity) OTUs from the ITS amplicon sequencing dataset (Supplementary Fig. 8f). All isolates belonged to Basidiomycota and Ascomycota, predominantly Dothideomycetes, Sordariomycetes, and Agaricomycetes (Fig. 5a–c), representing 15 total fungal orders. The white rot fungus *Irpex* cf. *lacteus* was detected in all wells by ITS amplicon sequencing, and cultured isolates were obtained for both *Irpex* cf. *lacteus* and an unclassified Helotiales species—two of the most ubiquitous taxa (Fig. 5e). In this study, 26 out of 67 culture groups could not be assigned to a genus, due to limited sequence similarity or ambiguous database matches, emphasizing that previously uncultured taxa can be successfully isolated. Well documented bias in ITS databases toward terrestrial fungi hampers marine fungal classification [98] and likely hampers subsurface identification as well. For future examination and description, a collection of cryopreserved and dried cultures, corresponding to all OTUs identified here, has been made publicly available at the University of Michigan Herbarium. Given historical limitations in subsurface fungal isolation, this constitutes the first public collection to date [22, 99]. Furthermore, integration of amplicon and geochemical data in this study enabled the reliable recovery of many dominant subsurface taxa, demonstrating a novel and effective approach for fungal isolation from deep subsurface waters.

### Candidate novel taxa

**Figure 6:**
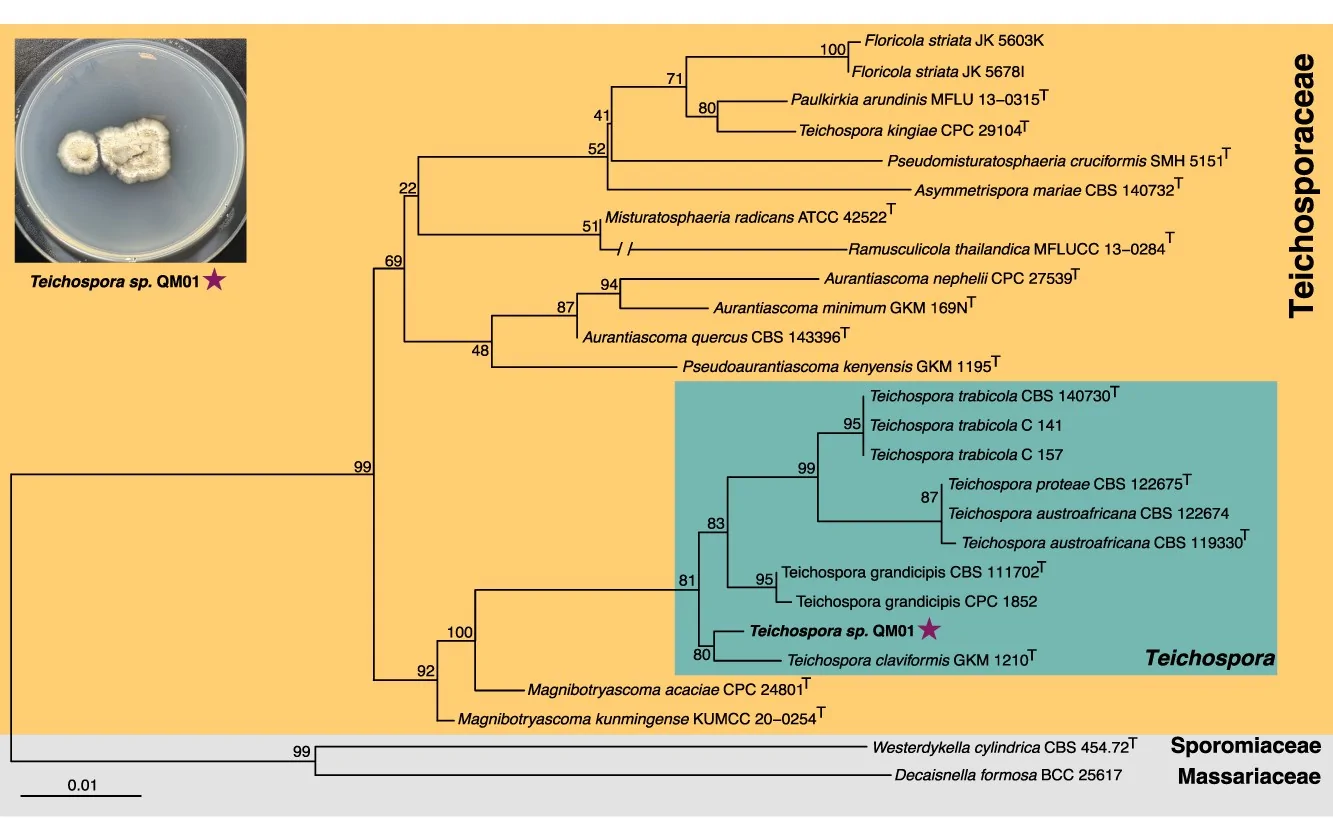
Maximum likelihood phylogram of Teichosporaceae inferred from 26 strains and a matrix of nrLSU sequences. The tree is rooted with *Westerdykella cylindrica* (CBS 454.72^T^) and *Decaisnella formosa* (BCC 25617) (Supplementary Table 3). Ex-type strains are denoted by ^T^. The focal candidate novel taxon (*Teichospora* sp. QM01) is shown in bold font and indicated with a purple star and photographed on PDA solid agar plate. Branch numbers show bootstrap support values; scale bar represents 0.01 expected changes per site.

Novelty assessment of fungal isolates involved comparing the newly obtained ITS sequences with the UNITE fungal reference, which includes both curated environmental DNA sequences and physical voucher specimens [48]. Thirteen cultured OTUs fell under the 98% similarity benchmark to known taxa, a common yet coarse threshold for delineating species [100]. Phylogenetic analysis of a unique isolate, *Teichospora* sp. QM01 (marked by an asterisk in Fig. 6), confirmed its clear separation from established *Teichospora* species. The closest phylogenetic relative, *Teichospora claviformis*, is recognized for its role in decomposing lignified plant material [101]. Strong phylogenetic support positions strain QM01 as either a previously unsequenced species or an uncharacterized lineage within Teichosporaceae (Ascomycota). While novel fungal lineages have been recovered in most surface habitats, this likely represents the first direct isolation of novel fungi from the terrestrial subsurface, particularly within organic carbon-rich formations.

Environmental sequencing often yields numerous unclassified 18S and ITS sequences, but the proportion of unclassified taxa is highly influenced by the choice of bioinformatic classifiers and the breadth of reference databases; thus, sequence data alone are insufficient to confirm true novelty [102, 103]. In this study, 13 potentially novel fungal taxa were successfully isolated, providing direct evidence that the subsurface Antrim Shale, and by extension subsurface habitats worldwide, harbor undescribed eukaryotic diversity. Fossil evidence indicates filamentous fungi have coexisted with bacteria in subsurface ecosystems for hundreds of millions of years, including in ~700-million-year-old shale [104], highlighting the potential significance of these environments in shaping global fungal evolution. Analogous findings in ancient mine waters led to the discovery of a new species of thermotolerant, asexual nematode [105]. Although global fungal diversity estimates vary widely, only ~5% have been formally described, and none account for subsurface biospheres [106]. Although evidence of fungal adaptation in terrestrial subsurface environments remains scarce, work on deep ocean sediments demonstrates unique survival strategies, such as novel ethanol production under anoxia [12, 27, 107]. As life persists in subsurface niches at depths exceeding 5 kilometers [19], these environments likely conceal diversity that may reveal evolutionary relationships, new metabolic pathways, and have biotechnological applications.

### Diverse eukaryotic communities among extremophilic methane producers

**Figure 7:**
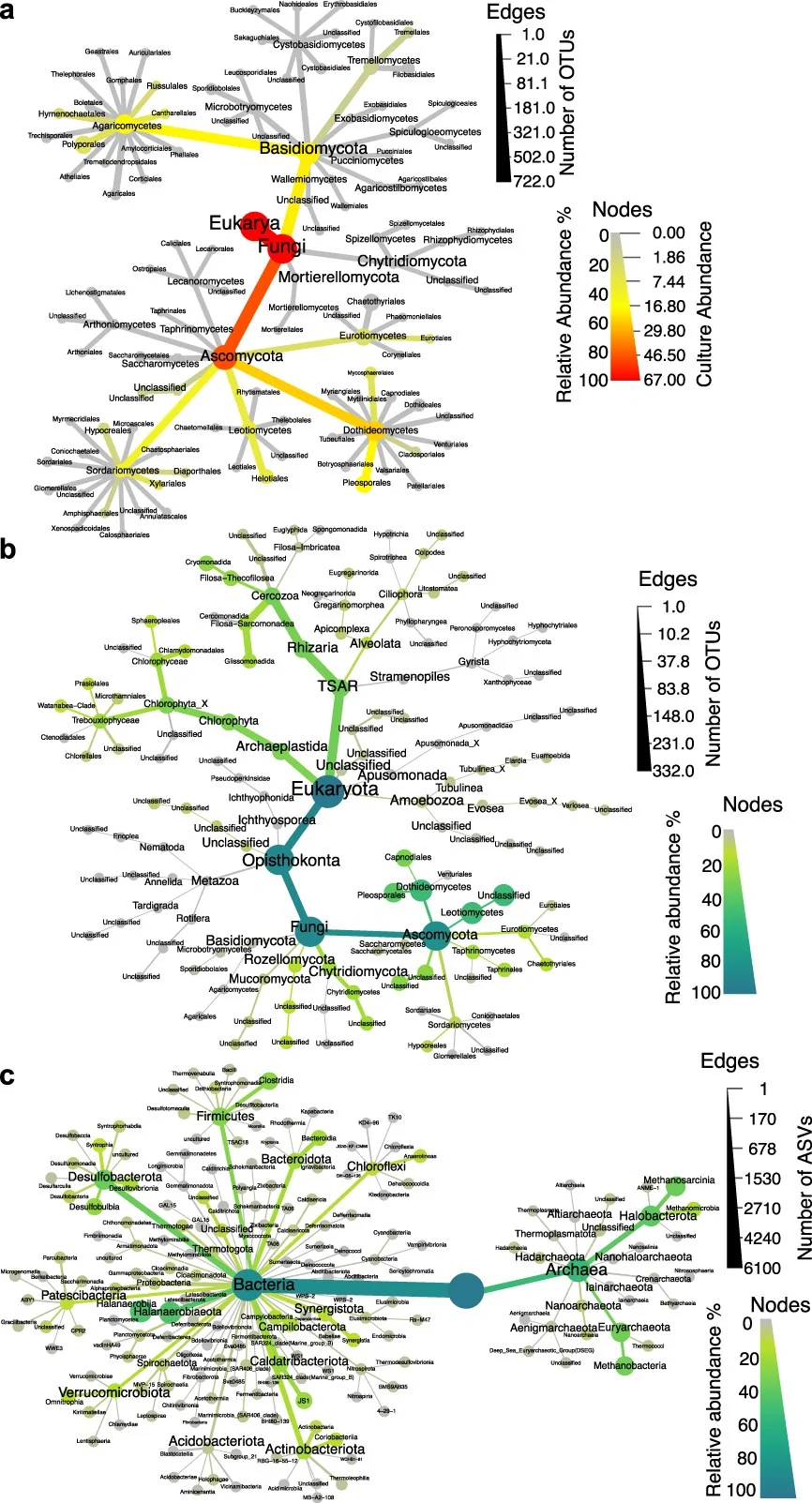
Heat trees illustrating the taxonomic composition and relative abundances within the core microbiome: (a) ITS, (b) 18S, and (c) 16S. The ITS heat tree (a) is further colored to reflect the abundance of cultured fungal OTUs at each node. For each primer, samples were rarefied to the minimum sequencing depth and then pooled across all sites to provide a snapshot of dominant community members. ITS and 18S heat trees are shown down to ordinal level, while the 16S heat tree is shown to the class level. These figures are tree-based visualizations of taxonomic classification and do not necessarily represent phylogenetic relationships.

Analysis of the bacterial and archaeal microbiome revealed a community composition indicative of a halophilic, methane-producing ecosystem in line with prior descriptions of the Antrim Shale [8, 33] (Fig. 7b). 16S rRNA gene sequencing identified 127 classes across 57 phyla, revealing pronounced bacterial dominance (68.9% of total reads). Prominent bacterial groups were Firmicutes, anaerobic halophilic Halanaerobiota, and sulfate-reducing Desulfobacterota. The archaeal community included representatives of three key methanogenic pathways and was dominated by halophilic taxa such as Euryarchaeota and Halobacterota.

**Figure 8:**
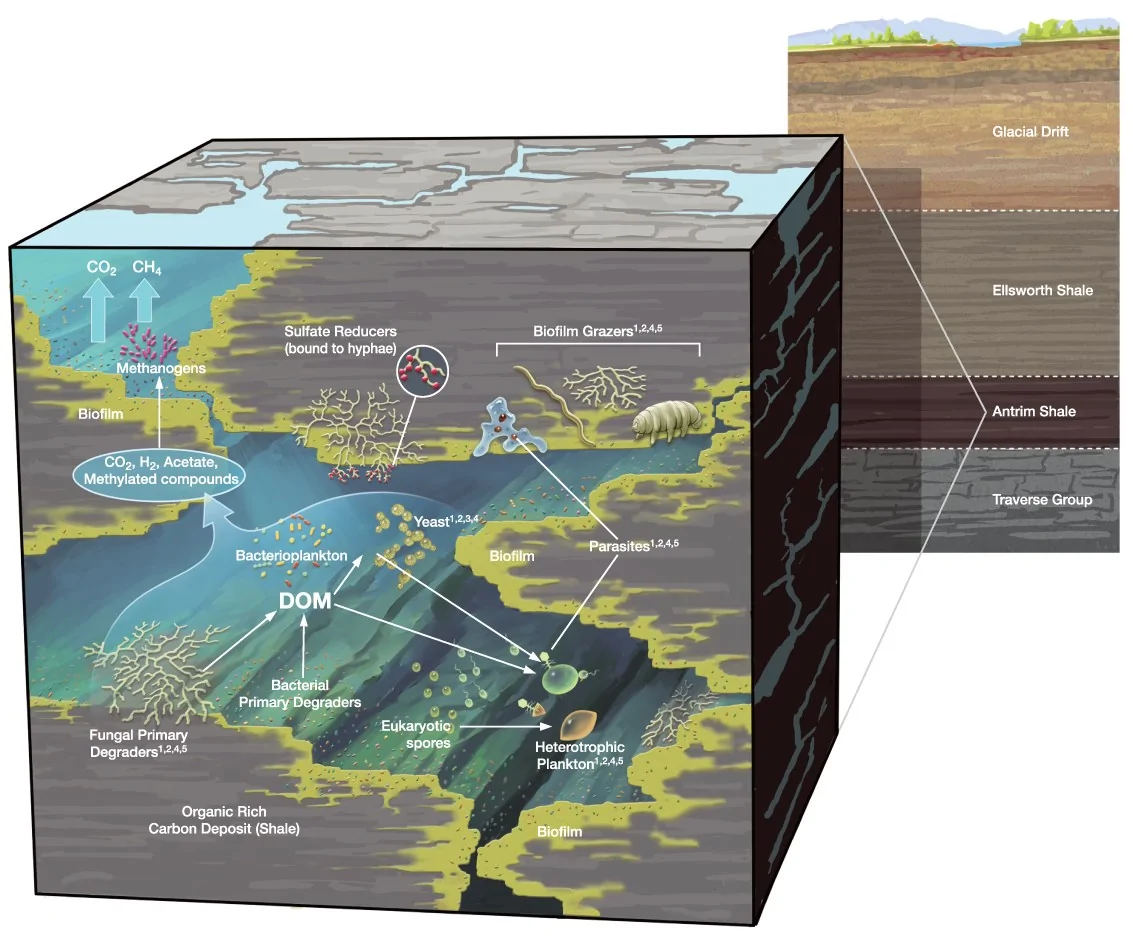
Conceptual food web and schematic overview illustrating the potential role of eukaryotes within organic-rich carbon deposit (Antrim shale) dependent subsurface ecosystems. Metabolites and compounds produced through eukaryotic activity are indicated by numbers (1–5): (1) dissolved organic material (DOM), (2) organic acids (e.g. lactate, acetate), (3) ethanol, (4) CO_2,_ (5) H_2_.

Fungal ITS sequencing of the same DNA extracts identified 689 OTUs across 20 classes, with 10.9% of reads unclassified at the family level (Fig. 7a). Sequence abundance must be interpreted with caution, as ribosomal gene copy number varies greatly across the tree of life, particularly in fungi where copy numbers can differ by up to two orders of magnitude [47, 108]. Agaricomycetes and Dothideomycetes were the most frequently detected classes in both culture-based and amplicon-based datasets, affirming their dominance (Fig. 7a). Although the two approaches showed substantial overlap in recovered fungal taxa, all isolation was performed under aerobic conditions, likely introducing bias toward aerobic and facultatively anaerobic taxa. 18S rRNA gene sequencing generally recovered similar fungal profiles and revealed additional diversity, with five fungal phyla and nine classes detected, including Rozellomycota and Mucoromycota, which were absent from the ITS dataset (Fig. 7b). 18S rRNA gene sequencing recovered just 95 fungal OTUs, with non-Dikarya fungi (e.g. Chytridiomycota) representing <1% of ITS reads, but up to 14% with the 18S marker. This primer bias underscores the importance of using multiple genetic markers to more accurately assess fungal richness and taxonomic diversity [109]. At phylum resolution, mycobiome profiles were consistent with previous subsurface studies [7, 110] and most other ecosystem surveys worldwide [111–113], predominantly comprised of Ascomycota and Basidiomycota and minor contributions from Mortierellomycota, Chytridiomycota, and other non-Dikarya phyla. Across both 18S and ITS approaches, yeasts were detected, comprising 12 OTUs within Saccharomycetes as well as genera such as *Sporobolomyces*, *Rhodotorula*, *Naganishia*, and *Filobasidium*. While fungi comprised 76.7% of the post-trimming 18S reads, the dataset also captured a broad eukaryotic spectrum spanning 21 classes within 8 microeukaryotic divisions. Sequences included representatives from Rhizaria, Chlorophyta, and metazoan groups such as Rotifera, Annelida, Tardigrada, and Nematoda (Fig. 7b) as well as intracellular parasites like Ichthyosporea, which are known known animal parasites [114], and Rozellomycota, which often parasitize other fungi [24]. Observed in both the culturable component and amplicon analysis, most eukaryotes were closely affiliated with surface taxa, with a fraction of novel lineages. A similar pattern is evident in marine environments [115, 116], where most marine taxa are nested within terrestrial lineages [113].

Given the inherent risk of contamination when sampling deep subsurface communities, human-associated taxa, such as *Malassezia* and common surface bacteria, were specifically screened for and not detected [29]. 18S rRNA gene analyses frequently identified plant DNA, particularly from *Pinus*, even in wells lacking evidence of recent surface recharge. The detection of photosynthetic taxa in deep subsurface samples is counterintuitive; however, similar findings have been reported in previous studies, including the widespread presence of green algae and cyanobacteria [6, 59, 117]. Although our study cannot directly determine the cause, similar observations are often attributed to ancient DNA [118], persistent hydrological connectivity, primer bias, or an expanded prevalence of heterotrophic metabolic strategies [6, 59, 117]. In contrast, both 16S rRNA gene analyses and geochemical profiles from this study indicate an isolated subsurface microbiome. Although active fungi have been detected in subsurface ecosystems [7, 115], substantial evidence suggests that many microorganisms in extreme environments may exist in a dormant rather than metabolically active state, and that detection does not uniformly indicate activity [119, 120]. Although this study focuses on a single geologic formation in one sedimentary basin, such site-specific investigations form the foundation of current knowledge in the subsurface [6, 28, 59, 89, 105, 121–123].

### Potential biogeochemical implications

While bacteria and archaea were once considered the sole mediators of oceanic organic matter degradation, fungi are now recognized as prevalent and, at times, dominant contributors to these processes [111]. Further, many organic carbon sources once thought too recalcitrant for biological degradation, including components of plastic, are now known to be susceptible to fungal decomposition in the environment [26, 124–126]. The similar absolute biomass, fungal:bacterial biomass ratio, and community composition between the examined formation waters and well-described marine settings, both aquatic systems, suggest that fungi and bacteria may analogously co-degrade recalcitrant carbon deposits. Our hydrogeologic context reveals that fossilized algal cells buried over 250 million years ago in the shale, rather than contemporary sinking phytoplankton, serve as the organic carbon input.

In subsurface environments, facultatively anaerobic fungi have already been implicated in the biogeochemical cycling of key elements, including nitrogen, phosphorus, iron, manganese, and sulfur, through mineral weathering and formation [127]. Fungal functions in subsurface organic-rich environments can broadly be categorized as shale-associated or planktonic. For shale-associated taxa, prior studies indicate that fungi support subsurface bacterial life by forming attached hyphal networks that facilitate symbiosis with sulfate-reducing bacteria through biogenic streams of H₂ [28]. Furthermore, by grazing on biofilm necromass, a niche also suspected for nematodes, facultatively anaerobic fungi may generate CO_2_, acetate, alcohols, and H₂ that directly fuel microbial methanogenesis [27, 127]. Here, we suggest that saprotrophic groups including numerous Dothideomycetes and Agaricomycetes taxa which are prevalent in the sampled wells and are extensively documented for transforming refractory and ancient organic and inorganic materials into bioavailable DOM and biomass at the surface [12, 15–17], perform similar functions in the subsurface (Fig. 8). Ascomycete and Basidiomycete fungi are documented to form associations with methanogenic archaea in anoxic bioreactors, collaboratively utilizing cellulose as a carbon source to generate methane [25]. In the Antrim Shale, preserved green algae, wood, and fractions of bitumen likely serve as carbon and energy sources susceptible to fungal decomposition even under anoxic conditions [128], as demonstrated by the use of polycyclic aromatic hydrocarbons (PAHs) in anoxic environments [12]. Accordingly, fungal release of carbon and energy from buried shale, through both direct biomass formation and the release of organic compounds, may not only drive methanogenesis, but also supply a constant stream of bioavailable organic carbon and energy that forms the foundation of the ecosystem and supports the persistence of complex microbial communities for thousands of years without surface inputs in the deep, isolated, and anoxic subsurface.

As an additional group, planktonic fungi, exemplified by the predominance of single cells (~99%) and detected yeasts, could be sustained by the high levels of measured DOC (2.2–52.6 mg C/L) in the formation waters. Although poorly understood, subsurface planktonic fungal communities could include spores, yeasts, dimorphic, and zoosporic forms. Zoosporic lineages have been documented as active participants in subsurface environments [7] especially in anaerobic decomposition of organic matter in buried sediments [124]. These abilities are perhaps linked to the evolutionary emergence of these lineages in low-oxygen aquatic environments [124]. Lastly, the occurrence of Rozellomycota in subsurface habitats along with the detection of various obligate intracellular parasites, can be explained by the presence of diverse fungal, metazoan, and protistan lineages that serve as susceptible hosts in the formation water. Collectively, these findings demonstrate that shale-derived carbon and energy support multitrophic, cross-domain food webs hundreds of meters below the surface, a process that has likely persisted for tens of thousands of years without ongoing surface input.

## Conclusions

Our findings challenge the common assumption that fungal regulation of organic matter decomposition is constrained to surface ecosystems. We show that fungi in the deep subsurface are not only present and diverse, as previously reported [6, 7, 11–13, 92], but can also substantially contribute to biomass, indicating an important role in subsurface carbon cycling. We propose that plant- and algal-associated saprobic and facultatively anaerobic fungal lineages, transported during periods of near-surface recharge, form a shale-associated fungal community, aligning with growing evidence that fungi can metabolize a broader range of recalcitrant organic carbon compounds and persist in environments historically considered inhospitable. Prolonged physical isolation over millennia, or tens of thousands of years in the examined Antrim Shale, may strongly select for diverse fungal lineages that are uncommon aboveground, as evidenced by the isolation of candidate novel taxa. These findings highlight the importance of including subsurface habitats in future assessments of global biodiversity. Ultimately, a more comprehensive understanding of the taxa and mechanisms involved in organic carbon conversion can refine predictive geochemical models and guide efforts to manage greenhouse gas emissions.

## Supplementary Material

Supplementary_material_wrag184

## Data Availability

Amplicon sequence data are publicly available under NCBI BioProject number PRJNA1346112. Representative vouchers for each cultured OTU were deposited in the University of Michigan Herbarium (MICH) under the catalog numbers 346816–346896 with representative strain sequences deposited into NCBI (Supplementary Table 2). Comprehensive geochemical data are available in the USGS data release and relevant data are also publicly available and downloadable on Zenodo (10.5281/zenodo.17883408).

## Code availability

All code necessary for reproduction of the results are publicly available via Github https://github.com/qmoon11/Eukaryote-Diversity-Antrim-Basin/tree/main.

## References

[1] Flemming H-C, Wuertz S. Bacteria and archaea on earth and their abundance in biofilms. *Nat Rev Microbiol* 2019;17:247–60. 10.1038/s41579-019-0158-9 30760902

[2] Hilton RG, West AJ. Mountains, erosion and the carbon cycle. *Nat Rev Earth Environ* 2020;1:284–99. 10.1038/s43017-020-0058-6

[3] Beaver RC, Neufeld JD. Microbial ecology of the deep terrestrial subsurface. *ISME J* 2024;18:wrae091. 10.1093/ismejo/wrae091 38780093 PMC11170664

[4] Pannekens M, Kroll L, Müller H, et al. Oil reservoirs, an exceptional habitat for microorganisms. *New Biotechnol* 2019;49:1–9. 10.1016/j.nbt.2018.11.006 PMC6323355 30502541

[5] Formolo MJ, Salacup JM, Petsch ST, et al. A new model linking atmospheric methane sources to Pleistocene glaciation via methanogenesis in sedimentary basins. *Geol* 2008;36:139. 10.1130/G24246A.1

[6] Westmeijer G, Van Dam F, Kietäväinen R, et al. Candidatus Desulforudis audaxviator dominates a 975 m deep groundwater community in central Sweden. *Commun Biol* 2024;7:1332. 10.1038/s42003-024-07027-2 39406897 PMC11480212

[7] Sohlberg E, Bomberg M, Miettinen H, et al. Revealing the unexplored fungal communities in deep groundwater of crystalline bedrock fracture zones in Olkiluoto, Finland. *Front Microbiol* 2015;6:6. 10.3389/fmicb.2015.00573 26106376 PMC4460562

[8] Martini AM, Walter LM, Budai JM, et al. Genetic and temporal relations between formation waters and biogenic methane: Upper Devonian Antrim Shale, Michigan Basin, USA. *Geochim Cosmochim Acta* 1998;62:1699–720. 10.1016/S0016-7037(98)00090-8

[9] McIntosh J, Kim J, Bailey L, et al. Burial and denudation alter microbial life at the bottom of the hypo-critical zone. *Geochem Geophys Geosyst* 2023;24:e2022GC010831. 10.1029/2022GC010831

[10] Schoell M . Multiple origins of methane in the earth. *Chem Geol* 1988;71:1–10. 10.1016/0009-2541(88)90101-5

[11] Han Q, Guo H, Zhang J, et al. Methane generation from anthracite by fungi and methanogen mixed flora enriched from produced water associated with the Qinshui Basin in China. *ACS Omega* 2021;6:31935–44. 10.1021/acsomega.1c04705 34870016 PMC8638023

[12] Zain Ul Arifeen M, Ma Y, Wu T, et al. Anaerobic biodegradation of polycyclic aromatic hydrocarbons (PAHs) by fungi isolated from anaerobic coal-associated sediments at 2.5 km below the seafloor. *Chemosphere* 2022;303:135062. 10.1016/j.chemosphere.2022.135062 35618067

[13] Jones DM, Head IM, Gray ND, et al. Crude-oil biodegradation via methanogenesis in subsurface petroleum reservoirs. *Nature* 2008;451:176–80. 10.1038/nature06484 18075503

[14] Strickland MS, Rousk J. Considering fungal:bacterial dominance in soils—methods, controls, and ecosystem implications. *Soil Biol Biochem* 2010;42:1385–95. 10.1016/j.soilbio.2010.05.007

[15] Li S, Li F, Li P, et al. Bio-solubilization of Yunnan lignite by Penicillium ortum MJ51 and characterization of its products. *Fuel* 2023;331:125923. 10.1016/j.fuel.2022.125923

[16] Wengel M, Kothe E, Schmidt CM, et al. Degradation of organic matter from black shales and charcoal by the wood-rotting fungus Schizophyllum commune and release of DOC and heavy metals in the aqueous phase. *Sci Total Environ* 2006;367:383–93. 10.1016/j.scitotenv.2005.12.012 16483638

[17] Haider R, Ghauri MA, SanFilipo JR, et al. Fungal degradation of coal as a pretreatment for methane production. *Fuel* 2013;104:717–25. 10.1016/j.fuel.2012.05.015

[18] Drake H, Ivarsson M. The role of anaerobic fungi in fundamental biogeochemical cycles in the deep biosphere. *Fungal Biol Rev* 2018;32:20–5. 10.1016/j.fbr.2017.10.001

[19] Magnabosco C, Lin L-H, Dong H, et al. The biomass and biodiversity of the continental subsurface. *Nat Geosci* 2018;11:707–17. 10.1038/s41561-018-0221-6

[20] McMahon S, Parnell J. Weighing the deep continental biosphere. *FEMS Microbiol Ecol* 2014;87:113–20. 10.1111/1574-6941.12196 23991863

[21] Ruff SE, Humez P, De Angelis IH, et al. Hydrogen and dark oxygen drive microbial productivity in diverse groundwater ecosystems. *Nat Commun* 2023;14:3194. 10.1038/s41467-023-38523-4 37311764 PMC10264387

[22] Ekendahl S, O’Neill AH, Thomsson E, et al. Characterisation of yeasts isolated from deep igneous rock aquifers of the Fennoscandian shield. *Microb Ecol* 2003;46:416–28. 10.1007/s00248-003-2008-5 14502418

[23] Whitman WB, Coleman DC, Wiebe WJ. Prokaryotes: the unseen majority. *Proc Natl Acad Sci* 1998;95:6578–83. 10.1073/pnas.95.12.6578 9618454 PMC33863

[24] Stüer-Patowsky K, Galindo LJ, Bösch Y, et al. Ecology of Rozellomycota in aquatic environments with differing redox conditions. *Fungal Biol* 2025;129:101670. 10.1016/j.funbio.2025.101670 41338735

[25] Chen R, Nie Y, Tanaka N, et al. Enhanced methanogenic degradation of cellulose-containing sewage via fungi-methanogens syntrophic association in an anaerobic membrane bioreactor. *Bioresou Technol* 2017;245:810–8. 10.1016/j.biortech.2017.09.046 28926913

[26] Lankiewicz TS, Choudhary H, Gao Y, et al. Lignin deconstruction by anaerobic fungi. *Nat Microbiol* 2023;8:596–610. 10.1038/s41564-023-01336-8 36894634 PMC10066034

[27] Zain Ul Arifeen M, Chu C, Yang X, et al. The anaerobic survival mechanism of Schizophyllum commune 20R-7-F01, isolated from deep sediment 2 km below the seafloor. *Environ Microbiol* 2021;23:1174–85. 10.1111/1462-2920.15332 33215844

[28] Drake H, Ivarsson M, Bengtson S, et al. Anaerobic consortia of fungi and sulfate reducing bacteria in deep granite fractures. *Nat Commun* 2017;8:55. 10.1038/s41467-017-00094-6 28676652 PMC5496868

[29] Rasmussen TB, Noell SE, Herbold CW, et al. Geothermal ecosystems on Mt. Erebus, Antarctica, support diverse and taxonomically novel biota. *FEMS Microbiol Ecol* 2024;100:fiae128. 10.1093/femsec/fiae128 39289026 PMC11523049

[30] Bueno de Mesquita CP, Vimercati L, Wu D, et al. Fungal diversity and function in metagenomes sequenced from extreme environments. *Fungal Ecol* 2024;72:101383. 10.1016/j.funeco.2024.101383

[31] Keeler E, Burgaud G, Teske A, et al. Deep-sea hydrothermal vent sediments reveal diverse fungi with antibacterial activities. *FEMS Microbiol Ecol* 2021;97. 10.1093/femsec/fiab103 34245561

[32] Stemple B, Tinker K, Sarkar P, et al. Biogeochemistry of the Antrim shale natural gas reservoir. *ACS Earth Space Chem* 2021;5:1752–61. 10.1021/acsearthspacechem.1c00087

[33] Wuchter C, Banning E, Mincer TJ, et al. Microbial diversity and methanogenic activity of Antrim shale formation waters from recently fractured wells. *Front Microbiol* 2013;4:4. 10.3389/fmicb.2013.00367 24367357 PMC3853793

[34] Martini AM, Walter LM, Ku TCW, et al. Microbial production and modification of gases in sedimentary basins: a geochemical case study from a Devonian shale gas play. *Michigan basin AAPG Bull* 2003;87:1355–75. 10.1306/031903200184

[35] McIntosh JC, Garven G, Hanor JS. Impacts of Pleistocene glaciation on large-scale groundwater flow and salinity in the Michigan Basin. *Geofluids* 2011;11:18–33. 10.1111/j.1468-8123.2010.00303.x

[36] Varonka MS, Barnhart EP, Tomaszewski EJ, et al. Geochemical data for produced water and gas from the Antrim Shale in Michigan, 2024. *US Geological Survey (USGS) Data Release* 2026;158. 10.5066/P1AUEAYP

[37] Gonzalez Y, Nelson DD, Shorter JH, et al. Precise measurements of^12^ CH_2_ D_2_ by tunable infrared laser direct absorption spectroscopy. *Anal Chem* 2019;91:14967–74. 10.1021/acs.analchem.9b03412 31663335

[38] Rodríguez-Ramos JA, Borton MA, McGivern BB, et al. Genome-resolved metaproteomics decodes the microbial and viral contributions to coupled carbon and nitrogen cycling in river sediments. *mSystems* 2022;7:e0051622–2. 10.1128/msystems.00516-22 35861508 PMC9426555

[39] White TJ, Bruns T, Lee S, Taylor J. Amplification and direct sequencing of fungal ribosomal RNA genes for phylogenetics. PCR Protocols*.* Academic Press, 1990, 315–22, 10.1016/B978-0-12-372180-8.50042-1.

[40] Liu CM, Aziz M, Kachur S, et al. BactQuant: an enhanced broad-coverage bacterial quantitative real-time PCR assay. *BMC Microbiol* 2012;12:56. 10.1186/1471-2180-12-56 22510143 PMC3464140

[41] Liu CM, Kachur S, Dwan MG, et al. FungiQuant: a broad-coverage fungal quantitative real-time PCR assay. *BMC Microbiol* 2012;12:255. 10.1186/1471-2180-12-255 23136846 PMC3565980

[42] Breyer E, Stix C, Kilker S, et al. The contribution of pelagic fungi to ocean biomass. *Cell* 2025;188:3992–4002.e13. 10.1016/j.cell.2025.05.004 40412391

[43] Ducklow H . Bacterial production and biomass in the oceans. In: Kirchman D.L. (ed.), Microbial Ecology of the Oceans, 2000, 85–120.

[44] Bolyen E, Rideout JR, Dillon MR, et al. Reproducible, interactive, scalable and extensible microbiome data science using QIIME 2. *Nat Biotechnol* 2019;37:852–7. 10.1038/s41587-019-0209-9 31341288 PMC7015180

[45] Callahan BJ, McMurdie PJ, Rosen MJ, et al. DADA2: high-resolution sample inference from Illumina amplicon data. *Nat Methods* 2016;13:581–3. 10.1038/nmeth.3869 27214047 PMC4927377

[46] Quast C, Pruesse E, Yilmaz P, et al. The SILVA ribosomal RNA gene database project: improved data processing and web-based tools. *Nucleic Acids Res* 2013;41:D590–6. 10.1093/nar/gks1219 23193283 PMC3531112

[47] Guillou L, Bachar D, Audic S, et al. The protist ribosomal reference database (PR2): a catalog of unicellular eukaryote small sub-unit rRNA sequences with curated taxonomy. *Nucleic Acids Res* 2013;41:D597–604. 10.1093/nar/gks1160 23193267 PMC3531120

[48] Abarenkov K, Nilsson RH, Larsson K-H, et al. The UNITE database for molecular identification and taxonomic communication of fungi and other eukaryotes: sequences, taxa and classifications reconsidered. *Nucleic Acids Res* 2024;52:D791–7. 10.1093/nar/gkad1039 37953409 PMC10767974

[49] Tedersoo L, Mikryukov V, Anslan S, et al. The global soil mycobiome consortium dataset for boosting fungal diversity research. *Fungal Divers* 2021;111:573–88. 10.1007/s13225-021-00493-7

[50] Tedersoo L, Bahram M, Zinger L, et al. Best practices in metabarcoding of fungi: from experimental design to results. *Mol Ecol* 2022;31:2769–95. 10.1111/mec.16460 35395127

[51] James TY, Stenlid J, Olson Å, et al. Evolutionary significance of imbalanced nuclear ratios in heterokaryons of Heterobasidion parviporum. *Evol* 2008;62:2279–96. 10.1111/j.1558-5646.2008.00462.x 18637961

[52] Fuller MS, Barshad I. Chitin and cellulose in the cell walls of Rhizidiomyces Sp. *Am J Bot* 1960;47:105–9. 10.1002/j.1537-2197.1960.tb07101.x

[53] Vilgalys R, Hester M. Rapid genetic identification and mapping of enzymatically amplified ribosomal DNA from several Cryptococcus species. *J Bacteriol* 1990;172:4238–46. 10.1128/jb.172.8.4238-4246.1990 2376561 PMC213247

[54] Kearse M, Moir R, Wilson A, et al. Geneious basic: an integrated and extendable desktop software platform for the organization and analysis of sequence data. *Bioinformatics* 2012;28:1647–9. 10.1093/bioinformatics/bts199 22543367 PMC3371832

[55] Katoh K, Asimenos G, Toh H. Multiple alignment of DNA sequences with MAFFT. In: Posada D (ed.), Bioinformatics for DNA Sequence Analysis. Totowa, NJ: Humana Press, 2009, 39–64, 10.1007/978-1-59745-251-9_3. 19378139

[56] Capella-Gutiérrez S, Silla-Martínez JM, Gabaldón T. trimAl: a tool for automated alignment trimming in large-scale phylogenetic analyses. *Bioinformatics* 2009;25:1972–3. 10.1093/bioinformatics/btp348 19505945 PMC2712344

[57] Nguyen L-T, Schmidt HA, von Haeseler A, et al. IQ-TREE: a fast and effective stochastic algorithm for estimating maximum-likelihood phylogenies. *Mol Biol Evol* 2015;32:268–74. 10.1093/molbev/msu300 25371430 PMC4271533

[58] R Core Team . R: A Language and Environment for Statistical Computing. Vienna, Austria. https://www.R-project.org/: R Foundation for Statistical Computing, 2024.

[59] Hubalek V, Wu X, Eiler A, et al. Connectivity to the surface determines diversity patterns in subsurface aquifers of the Fennoscandian shield. *ISME J* 2016;10:2447–58. 10.1038/ismej.2016.36 27022994 PMC5030689

[60] Meyer J, Zakhary S, Larocque M, et al. From surface to subsurface: diversity, composition, and abundance of sessile and endolithic bacterial, archaeal, and eukaryotic communities in sand, clay and rock substrates in the Laurentians (Quebec, Canada). *Microorganisms* 2022;10:129. 10.3390/microorganisms10010129 35056578 PMC8781179

[61] Lollar GS, Warr O, Telling J, et al. ‘Follow the water’: hydrogeochemical constraints on microbial investigations 2.4 km below surface at the Kidd Creek deep fluid and deep life observatory. *Geomicrobiol J* 2019;36:859–72. 10.1080/01490451.2019.1641770

[62] Craig H . Isotopic variations in meteoric waters. *Science* 1961;133:1702–3. 10.1126/science.133.3465.1702 17814749

[63] McIntosh JC, Walter LM. Paleowaters in Silurian–Devonian carbonate aquifers: geochemical evolution of groundwater in the Great Lakes region since the late Pleistocene. *Geochim Cosmochim Acta* 2006;70:2454–79. 10.1016/j.gca.2006.02.002

[64] US Department of Energy Office of Scientific and Technical Information Wilson TP. Origin and geochemical evolution of the Michigan basin brine (OSTI Report No. 6029640). 1989, https://www.osti.gov/biblio/6029640.

[65] Whiticar MJ . Carbon and hydrogen isotope systematics of bacterial formation and oxidation of methane. *Chem Geol* 1999;161:291–314. 10.1016/S0009-2541(99)00092-3

[66] Sherwood OA, Schwietzke S, Arling VA, et al. Global inventory of gas geochemistry data from fossil fuel, microbial and burning sources, version 2017. *Earth System Science Data* 2017;9:639–56. 10.5194/essd-9-639-2017

[67] Stolper DA, Lawson M, Davis CL, et al. Formation temperatures of thermogenic and biogenic methane. *Science* 2014;344:1500–3. 10.1126/science.1254509 24970083

[68] Martini AM, Walter LM, McIntosh JC. Identification of microbial and thermogenic gas components from Upper Devonian black shale cores. *Illinois and Michigan basins AAPG Bull* 2008;92:327–39. 10.1306/10180706037

[69] McIntosh JC, Walter LM, Martini AM. Extensive microbial modification of formation water geochemistry: case study from a midcontinent sedimentary basin. *United States GSA Bull* 2004;116:743–59. 10.1130/B25371.1

[70] Ferguson G, McIntosh JC, Grasby SE, et al. The persistence of brines in sedimentary basins. *Geophys Res Let* 2018;45:4851–8. 10.1029/2018GL078409

[71] McIntosh JC, Walter LM. Volumetrically significant recharge of Pleistocene glacial meltwaters into epicratonic basins: constraints imposed by solute mass balances. *Chem Geol* 2005;222:292–309. 10.1016/j.chemgeo.2005.07.010

[72] Mansour A, Adeyilola A, Gentzis T, et al. Depositional setting and organic matter characterization of the Upper Devonian Antrim Shale, Michigan Basin: implications for hydrocarbon potential. *Mar Pet Geol* 2022;140:105683. 10.1016/j.marpetgeo.2022.105683

[73] Strąpoć D, Mastalerz M, Dawson K, et al. Biogeochemistry of microbial coal-bed methane. *Annu Rev Earth Planet Sci* 2011;39:617–56. 10.1146/annurev-earth-040610-133343

[74] Kroeger KF, Di Primio R, Horsfield B. Atmospheric methane from organic carbon mobilization in sedimentary basins—the sleeping giant? *Earth-Sci Rev* 2011;107:423–42. 10.1016/j.earscirev.2011.04.006

[75] Long PE, Williams KH, Hubbard SS, et al. Microbial metagenomics reveals climate-relevant subsurface biogeochemical processes. *Trends Microbiol* 2016;24:600–10. 10.1016/j.tim.2016.04.006 27156744

[76] He L, Mazza Rodrigues JL, Soudzilovskaia NA, et al. Global biogeography of fungal and bacterial biomass carbon in topsoil. *Soil Biol Biochem* 2020;151:108024. 10.1016/j.soilbio.2020.108024

[77] Yergeau E, Bokhorst S, Huiskes AHL, et al. Size and structure of bacterial, fungal and nematode communities along an Antarctic environmental gradient. *FEMS Microbiol Ecol* 2007;59:436–51. 10.1111/j.1574-6941.2006.00200.x 16978243

[78] Taylor JD, Cunliffe M. Multi-year assessment of coastal planktonic fungi reveals environmental drivers of diversity and abundance. *ISME J* 2016;10:2118–28. 10.1038/ismej.2016.24 26943623 PMC4989315

[79] Richards TA, Leonard G, Mahé F, et al. Molecular diversity and distribution of marine fungi across 130 European environmental samples. *Proc Biol Sci* 2015;282:20152243. 10.1098/rspb.2015.2243 26582030 PMC4685826

[80] Hassett BT, Borrego EJ, Vonnahme TR, et al. Arctic marine fungi: biomass, functional genes, and putative ecological roles. *ISME J* 2019;13:1484–96. 10.1038/s41396-019-0368-1 30745572 PMC6775997

[81] Wang Y, Sen K, He Y, et al. Impact of environmental gradients on the abundance and diversity of planktonic fungi across coastal habitats of contrasting trophic status. *Sci Total Environ* 2019;683:822–33. 10.1016/j.scitotenv.2019.05.204 31154160

[82] Durkin CA, Mock T, Armbrust EV. Chitin in diatoms and its association with the cell wall. *Eukaryot Cell* 2009;8:1038–50. 10.1128/ec.00079-09 19429777 PMC2708456

[83] Gutiérrez M, Vera J, Srain B, et al. Biochemical fingerprints of marine fungi: implications for trophic and biogeochemical studies. *Aquat Microb Ecol* 2020;84:75–90. 10.3354/ame01927

[84] Rasconi S, Jobard M, Jouve L, et al. Use of calcofluor white for detection, identification, and quantification of phytoplanktonic fungal parasites. *Appl Environ Microbiol* 2009;75:2545–53. 10.1128/AEM.02211-08 19233954 PMC2675195

[85] Priest T, Fuchs B, Amann R, et al. Diversity and biomass dynamics of unicellular marine fungi during a spring phytoplankton bloom. *Environ Microbiol* 2021;23:448–63. 10.1111/1462-2920.15331 33201558

[86] De Vries FT, Hoffland E, Van Eekeren N, et al. Fungal/bacterial ratios in grasslands with contrasting nitrogen management. *Soil Biol Biochem* 2006;38:2092–103. 10.1016/j.soilbio.2006.01.008

[87] Westmeijer G, Turner S, Hevele P, et al. Exploring microbial diversity using cell-size fractionated enrichment incubations from subsurface aquifers at Äspö. *Sweden Commun Biol* 2026;9:378. 10.1038/s42003-026-09706-8 41691100 PMC12992704

[88] Wang X, Zhang W, Shao Y, et al. Fungi to bacteria ratio: historical misinterpretations and potential implications. *Acta Oecol* 2019;95:1–11. 10.1016/j.actao.2018.10.003

[89] Alfreider A, Krössbacher M, Psenner R. Groundwater samples do not reflect bacterial densities and activity in subsurface systems. *Water Res* 1997;31:832–40. 10.1016/S0043-1354(96)00311-9

[90] Bar-On YM, Phillips R, Milo R. The biomass distribution on earth. *Proc Natl Acad Sci* 2018;115:6506–11. 10.1073/pnas.1711842115 29784790 PMC6016768

[91] Barnhart EP, De León KB, Ramsay BD, et al. Investigation of coal-associated bacterial and archaeal populations from a diffusive microbial sampler (DMS). *Int J Coal Geol* 2013;115:64–70. 10.1016/j.coal.2013.03.006

[92] Nuppunen-Puputti M, Kietäväinen R, Purkamo L, et al. Rock surface fungi in deep continental biosphere—exploration of microbial community formation with subsurface *in situ* biofilm trap. *Microorganisms* 2020;9:64. 10.3390/microorganisms9010064 33383728 PMC7824546

[93] Carlson CA, Hansell DA, Nelson NB, et al. Dissolved organic carbon export and subsequent remineralization in the mesopelagic and bathypelagic realms of the North Atlantic basin. *Deep-Sea Res II Top Stud Oceanogr* 2010;57:1433–45. 10.1016/j.dsr2.2010.02.013

[94] Ge T, Luo C, Ren P, et al. Dissolved organic carbon along a meridional transect in the western North Pacific Ocean: distribution, variation and controlling processes. *Front Mar Sci* 2022;9:9. 10.3389/fmars.2022.909148

[95] Hansell DA, Carlson CA, Repeta DJ, et al. Dissolved organic matter in the ocean: new insights stimulated by a controversy. *Oceanography* 2009;22:202–11. 10.5670/oceanog.2009.109

[96] Shen Y, Benner R. Molecular properties are a primary control on the microbial utilization of dissolved organic matter in the ocean. *Limnol Oceanogr* 2020;65:1061–71. 10.1002/lno.11369

[97] Glassman SI, Martiny JBH. Broadscale ecological patterns are robust to use of exact sequence variants versus operational taxonomic units. *mSphere* 2018;3. 10.1128/msphere.00148-18 PMC6052340 30021874

[98] Peng X, Valentine DL. Diversity and N2O production potential of fungi in an oceanic oxygen minimum zone. *J Fungi* 2021;7:218. 10.3390/jof7030218 PMC8002691 33802893

[99] Liu C-H, Huang X, Xie TN, et al. Exploration of cultivable fungal communities in deep coal-bearing sediments from ∼1.3 to 2.5 km below the ocean floor. *Environ Microbiol* 2017;19:803–18. 10.1111/1462-2920.13653 28028923

[100] Tedersoo L, Mikryukov V, Zizka A, et al. Global patterns in endemicity and vulnerability of soil fungi. *Glob Change Biol* 2022;28:6696–710. 10.1111/gcb.16398 PMC9826061 36056462

[101] Tennakoon DS, Jeewon R, Thambugala KM, et al. Biphasic taxonomic approaches for generic relatedness and phylogenetic relationships of Teichosporaceae. *Fungal Divers* 2021;110:199–241. 10.1007/s13225-021-00492-8

[102] Poli A, Zanellati A, Piano E, et al. Cultivable fungal diversity in two karstic caves in Italy: under-investigated habitats as source of putative novel taxa. *Sci Rep* 2024;14:4164. 10.1038/s41598-024-54548-1 38378919 PMC10879487

[103] Fray D, McGovern CA, Casamatta DA, et al. Metabarcoding reveals unique microbial mat communities and evidence of biogeographic influence in low-oxygen, high-sulfur sinkholes and springs. *Ecol Evol* 2024;14:e11162. 10.1002/ece3.11162 38529029 PMC10961586

[104] Bonneville S, Delpomdor F, Préat A, et al. Molecular identification of fungi microfossils in a Neoproterozoic shale rock. *Sci Adv* 2020;6:eaax7599. 10.1126/sciadv.aax7599 32010783 PMC6976295

[105] Borgonie G, García-Moyano A, Litthauer D, et al. Nematoda from the terrestrial deep subsurface of South Africa. *Nature* 2011;474:79–82. 10.1038/nature09974 21637257

[106] Hawksworth DL, Lücking R. Fungal diversity revisited: 2.2 to 3.8 million species. *Microbiol Spectrum* 2017;5:10. 10.1128/microbiolspec.FUNK-0052-2016 PMC11687528 28752818

[107] Sobol MS, Hoshino T, Delgado V, et al. Genome characterization of two novel deep-sea sediment fungi, Penicillium pacificagyrus sp. nov. and Penicillium pacificasedimenti sp. nov., from South Pacific Gyre subseafloor sediments, highlights survivability. *BMC Genom* 2023;24:249. 10.1186/s12864-023-09320-6 PMC10173653 37165355

[108] Lofgren LA, Uehling JK, Branco S, et al. Genome-based estimates of fungal rDNA copy number variation across phylogenetic scales and ecological lifestyles. *Mol Ecol* 2019;28:721–30. 10.1111/mec.14995 30582650

[109] Tedersoo L, Anslan S, Bahram M, et al. Shotgun metagenomes and multiple primer pair-barcode combinations of amplicons reveal biases in metabarcoding analyses of fungi. *MC* 2015;10:1–43. 10.3897/mycokeys.10.4852

[110] Saitoh Y, Hirano S, Nagaoka T, et al. Genetic survey of indigenous microbial eukaryotic communities, mainly fungi, in sedimentary rock matrices of deep terrestrial subsurface. *Ecol Genet Genom* 2019;12:100042. 10.1016/j.egg.2019.100042

[111] Peng X, Amend AS, Baltar F, et al. Planktonic marine fungi: a review. *J Geophys Res G Biogeosciences* 2024;129:e2023JG007887. 10.1029/2023JG007887

[112] Egidi E, Delgado-Baquerizo M, Plett JM, et al. A few Ascomycota taxa dominate soil fungal communities worldwide. *Nat Commun* 2019;10:2369. 10.1038/s41467-019-10373-z 31147554 PMC6542806

[113] Gladfelter AS, James TY, Amend AS. Marine fungi. *Curr Biol* 2019;29:R191–5. 10.1016/j.cub.2019.02.009 30889385

[114] Shabardina V, Dharamshi JE, Ara PS, et al. Ichthyosporea: a window into the origin of animals. *Commun Biol* 2024;7:915. 10.1038/s42003-024-06608-5 39075159 PMC11286789

[115] Quemener M, Mara P, Schubotz F, et al. Meta-omics highlights the diversity, activity and adaptations of fungi in deep oceanic crust. *Environ Microbiol* 2020;22:3950–67. 10.1111/1462-2920.15181 32743889

[116] Ruff SE, De Angelis IH, Mullis M, et al. A global comparison of surface and subsurface microbiomes reveals large-scale biodiversity gradients, and a marine-terrestrial divide. *Sci Adv* 2024;10:eadq0645. 10.1126/sciadv.adq0645 39693444 PMC11654699

[117] Puente-Sánchez F, Arce-Rodríguez A, Oggerin M, et al. Viable cyanobacteria in the deep continental subsurface. *Proc Natl Acad Sci* 2018;115:10702–7. 10.1073/pnas.1808176115 30275328 PMC6196553

[118] Wegner C-E, Stahl R, Velsko I, et al. A glimpse of the paleome in endolithic microbial communities. *Microbiome* 2023;11:210. 10.1186/s40168-023-01647-2 37749660 PMC10518947

[119] Bradley JA . Microbial dormancy as an ecological and biogeochemical regulator on earth. *Nat Commun* 2025;16:3909. 10.1038/s41467-025-59167-6 40280922 PMC12032139

[120] Wörmer L, Hoshino T, Bowles MW, et al. Microbial dormancy in the marine subsurface: global endospore abundance and response to burial. *Sci Adv* 2019;5:eaav1024. 10.1126/sciadv.aav1024 30801015 PMC6382399

[121] Kadnikov VV, Mardanov AV, Beletsky AV, et al. Phylogeny and physiology of candidate phylum BRC1 inferred from the first complete metagenome-assembled genome obtained from deep subsurface aquifer. *Syst and Appl Microbiol* 2019;42:67–76. 10.1016/j.syapm.2018.08.013 30201528

[122] Borgonie G, Linage-Alvarez B, Ojo AO, et al. Eukaryotic opportunists dominate the deep-subsurface biosphere in South Africa. *Nat Commun* 2015;6:8952. 10.1038/ncomms9952 26597082 PMC4673884

[123] Bengtson S, Rasmussen B, Ivarsson M, et al. Fungus-like mycelial fossils in 2.4-billion-year-old vesicular basalt. *Nat Ecol Evol* 2017;1:0141. 10.1038/s41559-017-0141 28812648

[124] Orsi WD, Vuillemin A, Coskun ÖK, et al. Carbon assimilating fungi from surface ocean to subseafloor revealed by coupled phylogenetic and stable isotope analysis. *ISME J* 2022;16:1245–61. 10.1038/s41396-021-01169-5 34893690 PMC9038920

[125] Guo K, Zhao Z, Breyer E, et al. Organic matter degradation by oceanic fungi differs between polar and non-polar waters. *Nat Commun* 2025;16:7589. 10.1038/s41467-025-63047-4 40817327 PMC12356891

[126] Vaksmaa A, Polerecky L, Dombrowski N, et al. Polyethylene degradation and assimilation by the marine yeast Rhodotorula mucilaginosa. *ISME Commun* 2023;3:68. 10.1038/s43705-023-00267-z 37423910 PMC10330194

[127] Ivarsson M., Bengtson S., Drake H., Francis W. Fungi in deep subsurface environments. Adv Appl Microbiol, 2018;102:83–116. 10.1016/bs.aambs.2017.11.001, San Diego, Calif., Elsevier. 29680127

[128] Kietäväinen R, Purkamo L. The origin, source, and cycling of methane in deep crystalline rock biosphere. *Front Microbiol* 2015;6. 10.3389/fmicb.2015.00725 PMC4505394 26236303

