# Supplementary material for: Deep subsurface organic-rich shale supports abundant, diverse, and novel fungi
Source: ISME J. 2026 Jul 29;20(1):wrag184. doi: 10.1093/ismejo/wrag184 (PMC13502201; doi:10.1093/ismejo/wrag184)
Supplement: Supplementary file 1 [file supplementary_material_wrag184.zip › ISMEJ_Supplementary_final.pdf]

Supplementary Material for:

Deep subsurface organic-rich shale supports abundant, diverse, and novel fungi

Quinn S. Moon[1,2], Elliott P. Barnhart[3], Matthew S. Varonka[4], Elizabeth J. Tomaszewski[4], Michelle Orozco-Quime[1], Thomas Desrosiers[1], Ivan Paciorka[1], Michael Carley[5], James Schramski[5], Bradley S. Stevenson[6], Magdalena R. Osburn[6], Anurup Mohanty[6], Anna M. Martini[7], Jason E. Stajich[8], Jennifer C. McIntosh[9], Timothy Y. James[1,10]

[1] Department of Ecology and Evolutionary Biology, University of Michigan, Ann Arbor, MI 48108, USA
[2] Institute for Global Change Biology, University of Michigan, Ann Arbor, MI 48108, USA
[3] U.S. Geological Survey, Wyoming-Montana Water Science Center, Helena, MT 59601, USA
[4] U.S. Geological Survey, Geology, Energy & Minerals Science Center, Reston, VA 20191, USA
[5] Riverside Energy Michigan, LLC, Traverse City, MI 49684, USA
[6] Department of Earth, Environmental, and Planetary Sciences, Northwestern University, Evanston, IL 60208, USA
[7] Geology Department, Amherst College, Amherst, MA 01002, USA
[8] Department of Microbiology & Plant Pathology and Institute for Integrative Genome Biology, University of California Riverside, Riverside, CA 92521, USA
[9] Department of Hydrology and Atmospheric Sciences, University of Arizona, Tucson, AZ 85721, United States
[10] University of Michigan Herbarium (MICH), Ann Arbor, MI 48108, USA

Table of Contents

Additional Methods Details

Fungal Isolation
Fungal Isolate Identification
DNA extractions, library preparation and sequencing
Enumeration Cell Counts
qPCR Relative Abundances
rRNA Sequence Processing
Community Analysis

Supplementary Figures

Figure 1. Representative enumeration FOVs
Figure 2. Species accumulation curves for technical replicates
Figure 3. Pooled species accumulation curves (by reads, by sample sites)
Figure 4. NMDS grouping of techinal replicates
Figure 5. Geochemistry by depth
Figure 6. Fungal richness vs. read count, vs 16S rRNA gene richness
Figure 7. Taxonomic stacked bar plots
Figure 8. Fungal isolate characteristics
Figure 9. Alpha diversity with 95% interval

Supplementary Tables

Table 1. Complete geochemistry and stable isotope values
Table 2. Strain ID, taxonomy, sequence similarity to UNITE ITS database, ITS sequence accession numbers, and University of Michigan Herbarium Voucher numbers of fungal isolates.
Table 3. Taxa names, strain ID, and accession numbers included in phylogenetic placement of candidate novel isolate.

Supplementary Data (not included in this document, available as downloadable .xlsx file)

Supplementary Data 1: Abundance (enumeration and qPCR) values by sampled well
Supplementary Data 2: Referenced Antrim Shale geochemical isotope values

Any use of trade, firm, or product names is for descriptive purposes only and does not imply endorsement by the U.S. Government.

All data are additionaly available on Zenodo (https://zenodo.org/records/17883408?token=eyJhbGciOiJIUzUxMiJ9.eyJpZCI6IjJhODIzNDgzLTg0NDYtNDNkMy1hZjYzLTRlMmVkMmVmNjdiMiIsImRhdGEiOnt9LCJyYW5kb20iOiIzY2YxMjc3MjQ3YTc3NjgyZGFhM2QzMWQ5YjYwNzExZiJ9.7fVVVTGiyUr_Ir3HAnoJihu-54ddXYvna9DzS7TU0x7HONRTtQ7o3NY1xGG2_0FjTvQqC-c-zaHc3glkC72V8g) or in the U.S. Geological Survey (USGS) Data Release [1].

Methods

Fungal Isolation

After filtering formation water, the 1.2 µm filters were placed in sterile double-distilled water (ddH_2O) and manually shaken vigorously to dislodge attached cells. A 500 µL aliquot of the resulting suspension was spread in five replicates onto each of two types of media: PDA and GY5. Potato dextrose agar (PDA) consisted of 24 g potato dextrose broth, 20g agar per liter of water. Dilute (1/10th) GY5 medium consisted if 1 (g) yeast extract, 0.3 (g) glucose, 1.4 (g) KH_2PO_4, 0.6 (g) Na_2HPO_4, 0.1 (g) $MgSO_4$, 20 (g) agar per liter of water. To suppress bacterial growth, all media were supplemented with Streptomycin sulfate (200 mg/L) and Penicillin G (200 mg/L). Axenic cultures were obtained by repeated transfer of colonies onto fresh plates of the same medium. Axenic cultures were preserved on PDA slants, dried PDA media plates, and in 30% glycerol at −80 °C. Fully grown colonies on PDA plates were dried for two days under ambient air conditions, and a representative dried agar plate along with a glycerol stock was deposited in the University of Michigan Herbarium.

Fungal Isolate Identification

DNA was extracted from all axenic fungal cultures by scraping ~300 mg of tissue from culture plates and using a CTAB protocol [2]. DNA concentrations were assessed via fluorometry using a Qubit high sensitivity kit (Thermo Fisher Scientific). The internal transcribed spacer (ITS) region including 5.8S rRNA gene (nrITS) was amplified using the primer pair ITS1/ITS4 [3]. Negative controls without DNA template were included in all polymerase chain reactions (PCR). Amplicons were visualized on a 1% agarose gel and purified using ExoSAP prior to Sanger sequencing in the forward direction (ITS1) at Genewiz (Genewiz Inc., South Plainfield, NJ, USA). Resulting ABI chromatograms were quality checked and trimmed using Geneious v10.1.2 [4]. To compare isolate sequences with amplicon sequencing data, the ITS isolate sequences were used to create a custom database for BLASTn searches against the amplicon sequence variants (ASVs). Isolates were classified as a match if their sequences exhibited ≥98% similarity to an amplicon sequence, as determined by pairwise alignment. Isolates meeting this criterion were clustered accordingly and assigned the taxonomic designation of the corresponding amplicon sequence. Sanger sequences not present in the amplicon dataset were clustered into 98% operational taxonomic units (OTUs) and classified using the pipeline described above.

DNA extractions, library preparation and sequencing

DNA isolation from frozen filters was carried out as previously described [5]. In short, phosphate-buffered saline (PBS) solution containing 0.1% Na pyrophosphate solution was used to release biomass from the filter and DNA was extracted from the resulting pellets using the QIAGEN PowerWater Kit (MoBio Laboratories, Inc., Carlsbad, CA) in accordance with the manufacturer's instructions, with an increased (2-h) proteinase K incubation at 55°C prior to bead beating to improve cell lysis. Due to high particulate shale load, filter samples from well Little Big Horn D4-4 were extracted using FastDNA SPIN Kit for Soil (MP Biomedicals), following the manufacturer's instructions. The quantity of extracted DNA was assessed via fluorometry using a Qubit high sensitivity kit. Extracted DNA was sent to Integrated Microbiome

Core (IMR) at Dalhousie University for gene amplification, library preparation, and paired end 2 × 300 bp Illumina MiSeq sequencing. The fungal ITS rRNA variable region was amplified using the forward primer (3'-GTGAATCATCGAATCTTTGAA-5') and reverse primer ITS4 (3'-TCCTCCGCTTATTGATATGC-5') [6]. The bacterial and archaeal 16S rRNA V4 variable gene region was amplified using the forward primer 515FB (3'-GTGYCAGCMGCCGCGGTAA-'5') and reverse primer 806RB (3'-GGACTACNVGGGTWTCTAAT-'5') [7]. The eukaryote 18S rRNA V4 variable gene region was amplified using the forward primer E572F (3'-CYGCGGTAATTCCAGCTC-'5') and reverse primer E1009R (3'-AYGGTATCTRATCRTCTTYG-'5') [8].

Enumeration Cell Counts

Microbial cell abundance was quantified using epifluorescence microscopy. For each sample previously fixed to a final concentration of 2% glutaraldehyde, 2 mL was placed in a 25 mm glass microanalysis filter holder (Millipore Sigma) containing a black polycarbonate filter (STERLITECH Polycarbonate, 1270059, 25 mm, 0.2 µm pore size) on top of a glass microfiber filter (Whatman glass microfiber grade GF/C filter discs, 25 mm) clamped onto a glass fritted support. The cells were deposited onto the filter via vacuum. For total cell counts, samples were stained with 1 mL of 4′,6-diamidino-2-phenylindole (DAPI) solution at 5 µg/mL. For enumerating fungal cells exclusively, replicate samples were stained using 1 mL of Calcofluor White (CFW) solution at 100 µg/mL. After incubation for 5 min in the dark, the stain solution was removed via filtration, followed by rinsing with 1 mL of sterile, filtered ddH_2O. Stained filters were sandwiched between a microscope slide and coverslip containing a thin film of immersion oil, and immediately examined using a Zeiss Axioscope Imager2 microscope (Carl Zeiss, Jena, Germany) at 1000× magnification and imaged using Zeiss Axiocam (506 mono). DAPI and CFW stained cells were visualized under UV excitation using Zeiss Filter set 49. For CFW staining, at least 30 randomly selected fields of view (FOVs) were counted, whereas for DAPI staining, random FOVs were examined until a total of 300 cells had been counted with at least 20 FOVs (representative FOVs shown in Supplementary Fig. 2). Only fungal cells with a diameter greater than 1 µm were counted.

qPCR Relative Abundances

The relative abundance of fungi to bacteria was quantified using the BactQuant TaqMan 16S rRNA gene assay [9] and the FungiQuant TaqMan 18S rRNA gene assay [10], employing previously described primers and probes, on a QuantStudio™3 machine Real-Time PCR system (Thermo Fisher Scientific). For each sample site, the filtration replicate yielding the highest DNA concentration was selected for qPCR analysis, and three independent qPCR assays were performed for each gene. The reactions were run with a volume of 10 µl consisting of 1 µl 18 µM forward primer, 1 µl 18 µM reverse primer, 1 µl 2.25 µM TaqMan probe, 1 µl BSA (400 ng/uL), 5 µl 2X master mix [Radiant Probe Lo-ROX qPCR Kit (Alkali Scientific QP9005),)] and 1 µl DNA template diluted to 0.1 ng/ul. The thermocycling conditions were as follows: polymerase activation at 95°C for 1 minute followed by 50 cycles of 95°C for 1 second and 55°C for 20 seconds. Abundance estimates were made relative to quantified synthetic *Escherichia coli* (strain U 5/41 16S rRNA) and *Saccharomyces cerevisiae* (strain NRRL Y-12632 18S rRNA gene, from type material) dsDNA standards (gBlocks) in serial dilutions ranging from 10^1 to 10^7 copies for

each gene. qPCR values were corrected to a genome counts basis using median ribosomal RNA operon copy number [11][12]. The qPCR efficiencies for both genes ranged between 99.2 and 99.7% and $R^2 > 0.94$. Cell ratios were converted to biomass ratios using the conversion factor of 10 fg C/ bacterial cell and 6,469 fg C/ fungal cell, as previously applied to pelagic bacterial and fungal populations [13][14].

Amplicon rRNA gene sequence processing

Primer sequences were removed from the dataset by the core facility, and subsequent verification confirmed that no primer sequences remained. Initial processing and quality control of demultiplexed raw rRNA gene amplicon data sequence data for all three primers sets (ITS2, 16S, 18S) were performed using QIIME 2 (v2021.11 81) [15]. Denoising and chimera removal was performed with DADA2 using default parameters with truncation cutoffs based on mean Phred scores dropping below 30 (250 bp (forward) and 150 bp (reverse) for 16S, 260 (forward) and 210 bp (reverse) for 18S, 210 (forward) and 170 bp (reverse) for ITS). Chimeric sequences and reads with more than two expected errors were removed. In the high diversity 16S dataset, the --p-min-fold-parent-over-abundance parameter was increased to 3 to reduce read loss from chimera filtering. Amplicon sequence variants (ASVs) were then clustered at 99%, and 98% identity using VSEARCH [16] for 18S and ITS respectively [17].

Taxonomy was assigned separately for each marker gene using approaches suitable for environments with potentially novel taxa [18]. Taxonomic classification of 16S rRNA gene sequences was performed using a naïve Bayes classifier trained on the SILVA database (v138, V4 region) [19]. For 18S rRNA gene and ITS data, OTU representative sequences were exported from QIIME and classified separately. 18S rRNA gene sequences were assigned taxonomy using the naïve Bayesian classifier implemented in DADA2 (minBoot = 80), against both the PR2 v5.1.0 [20] and SILVA v138 databases [19]. Assignments to fungi versus microeukaryotes were in complete agreement for both databases. Therefore, PR2 assignments were used for microeukaryotes, while SILVA assignments were used for fungi. For ITS sequences, a BLASTn based approach was used against the general eukaryotic UNITE v10.0 database [21], as recommended for environments containing undescribed fungal taxa [18]. Taxonomic assignments were based on the top 10 BLAST hits and evaluated using taxon specific e-values and sequence similarity thresholds [18] [22]. The resulting ASV/OTU tables, representative sequences, and taxonomy were imported into R v4.3.1 [23] for further analysis. For ITS2 fungal analyses, all non-fungal sequences were excluded (5.4 %). In the 18S rRNA gene dataset, OTUs classified as land plants (Division = Streptophyta), vertebrates (Class = Craniata), or arthropods (Class = Arthropoda) were dropped from analysis and accounted for 64.5%, 0.26%, and <0.01% of reads and 49, 1, and 1 OTUs, respectively. Fungal OTUs identified in the 18S data were grouped as '18S Fungi,' while all remaining taxa were classified as '18S Microeukaryotes.'

Samples containing fewer than 320 quality trimmed 18S rRNA gene reads or fewer than 300 quality trimmed ITS reads were excluded from downstream analyses, whereas all 16S rRNA gene samples were retained. After trimming, 3 of the sample sites failed to have a single replicate with greater than 320 18S reads and these sites were dropped from downstream analysis. Non-metric multidimensional scaling (NMDS) was employed to evaluate the reproducibility of replicates within each well using the R [23] package *vegan[24],* using the function *metaMDS* [24]. Replicates demonstrated strong clustering by sample site (Supplementary Fig. 3) and were thus pooled for downstream analyses.

Community and Statistical Analyses

To analyze and visualize the archael, bacterial, fungal, and microeukartic composition, we used various packages within R [23], with *ggplot2* [25] used for all visualization. Stacked bar plots were assembled using *phyloseq* [26], community analyses and diversity metrics (Observed richness, Chao1 richness, Shannon diversity index, Hurlbert's PIE evenness) were calculated using *vegan* [24]. Investigations of the core microbiome were conducted and visualized with *Metacoder* [27]. For alpha diversity analyses, we chose not to rely on rarefaction approach because sequencing depth was sufficient to capture diversity without subsampling. For distance–decay and core microbiome analyses, however, we rarefied to the minimum read count per sample to ensure comparability across samples using *vegan* [24]. Statistical relationships were evaluated using Spearman's rank correlations (cor.test) and linear models (lm) implemented in the base R *stats* package [23].

Supplementary Figures

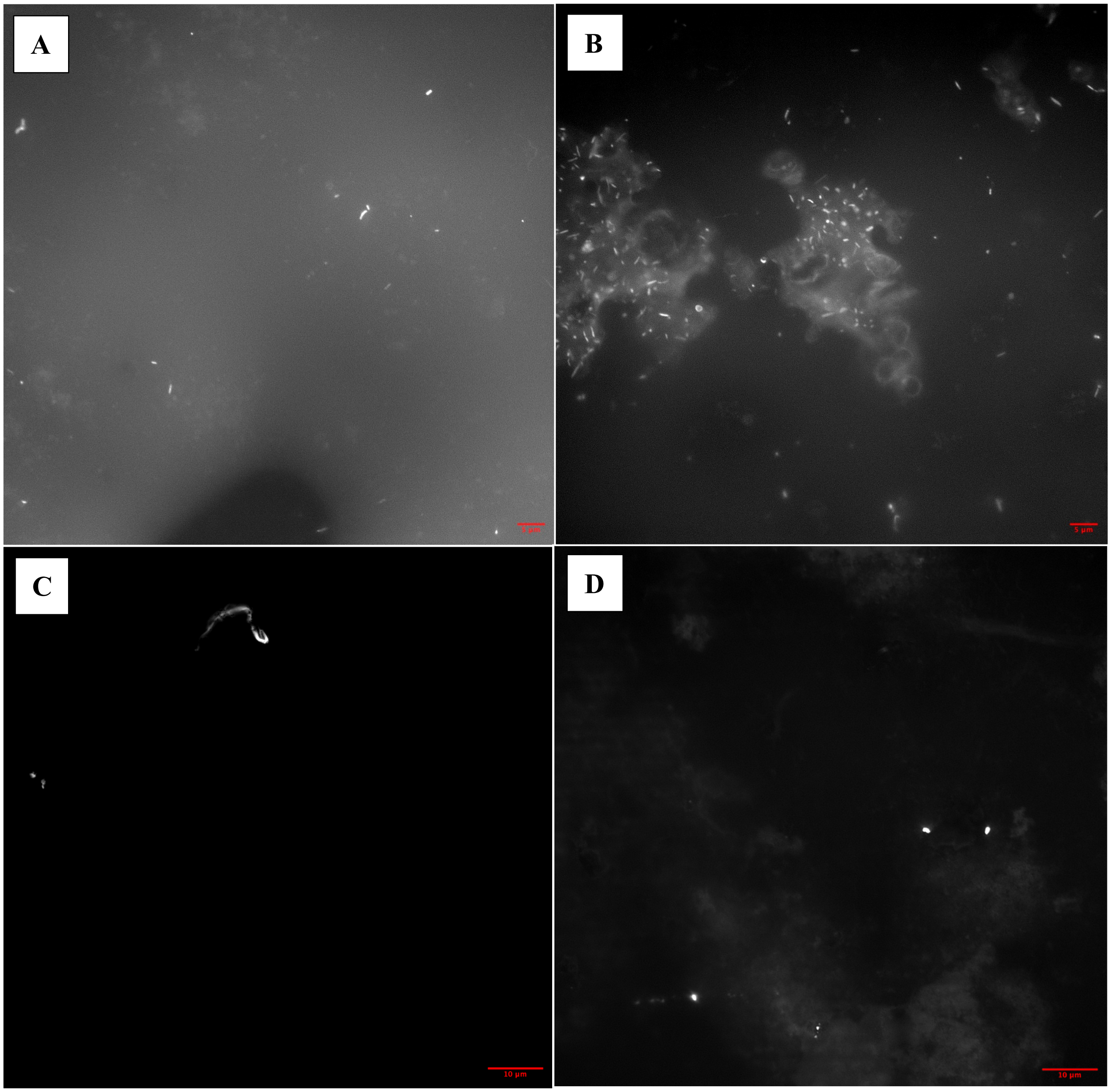

Supplementary Figure 1. Representative microscopic enumeration field of views (FOVs) from (A-B) DAPI-stained and (C-D) Calcofluor White (CFW) stained samples, shown with relevant scale bar. DAPI-stained cells were used to quantify total microbial abundance, while CFW stained cells were used exclusively to quantify fungal abundance in the formation water samples.

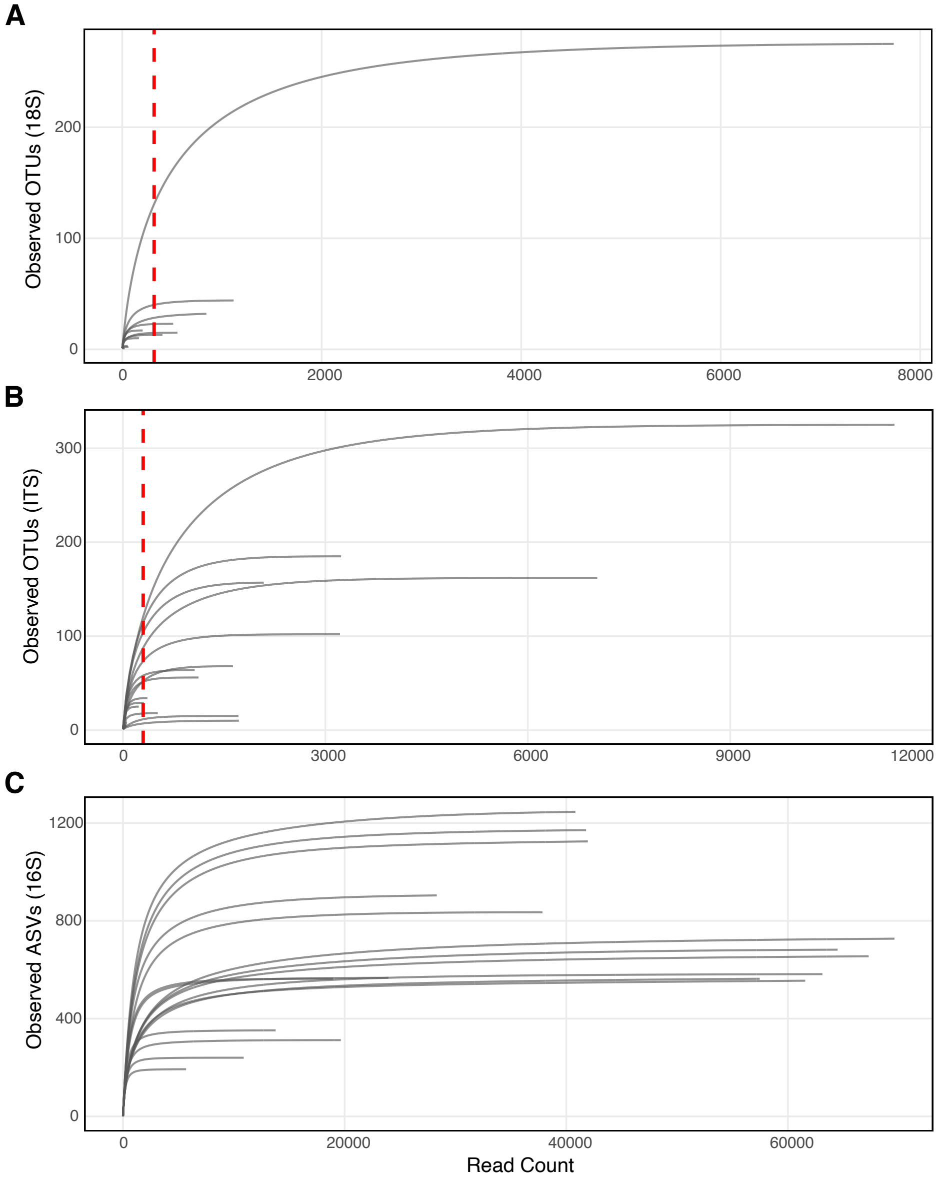

Supplementary Figure 2. Amplicon sequent variant (ASV) and operational taxonomic unit (OTU) accumulation curves for each technical replicate prior to sample pooling: (A) 18S rRNA gene, (B) ITS region, and (C) 16S rRNA gene. Minimum read-count cutoff values applied to the 18S and ITS datasets are indicated by vertical red dashed lines. No cutoff was applied to the 16S rRNA gene sequences, as all samples reached saturation.

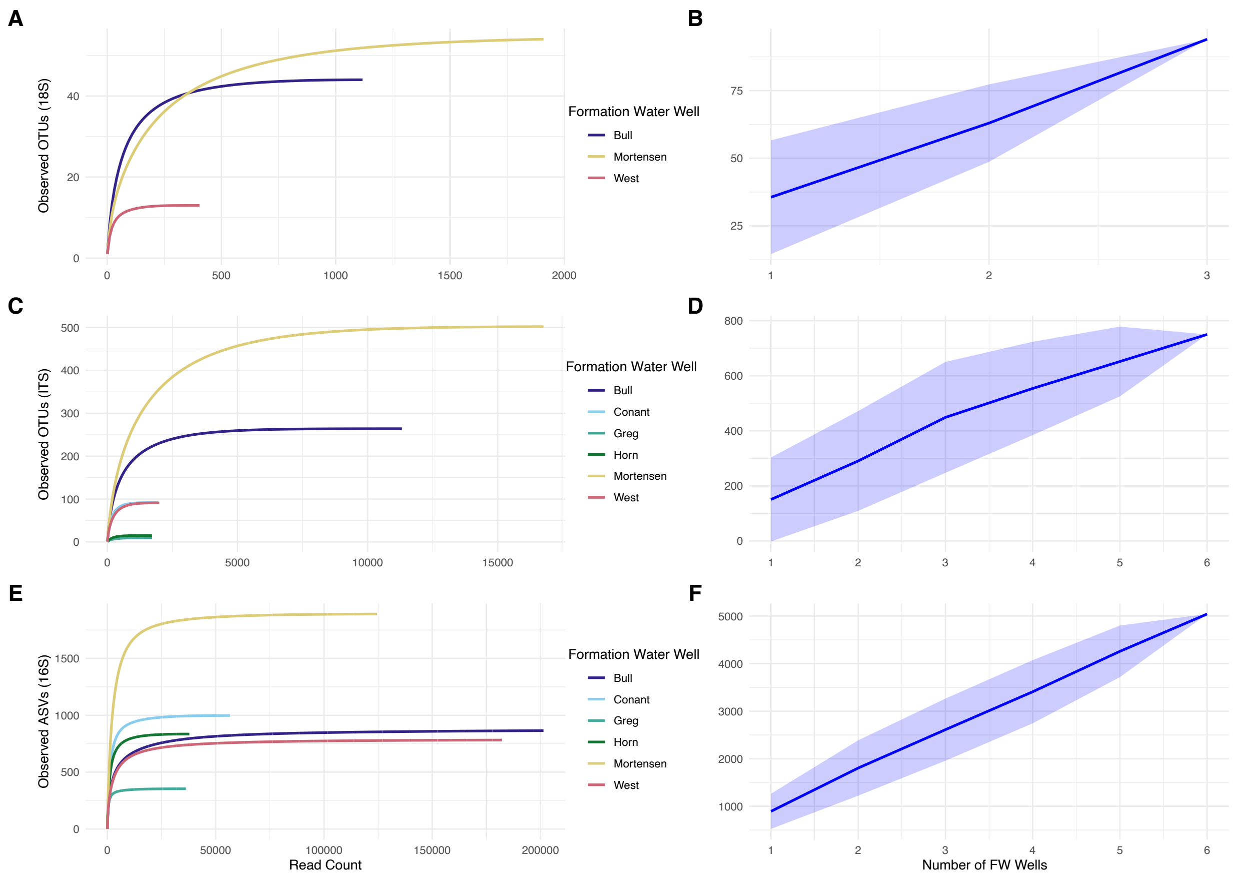

Supplementary Figure 3. Amplicon sequent variant (ASV) and operational taxonomic unit (OTU) accumulation curves for included Illumina reads and samples: (A–B) 18S rRNA gene, (C–D) ITS, and (E–F) 16S rRNA gene. Accumulation curves represent pooled reads from each technical replicate per sampled formation water well (FW), following non-metric multidimensional scaling (NMDS) inspection.

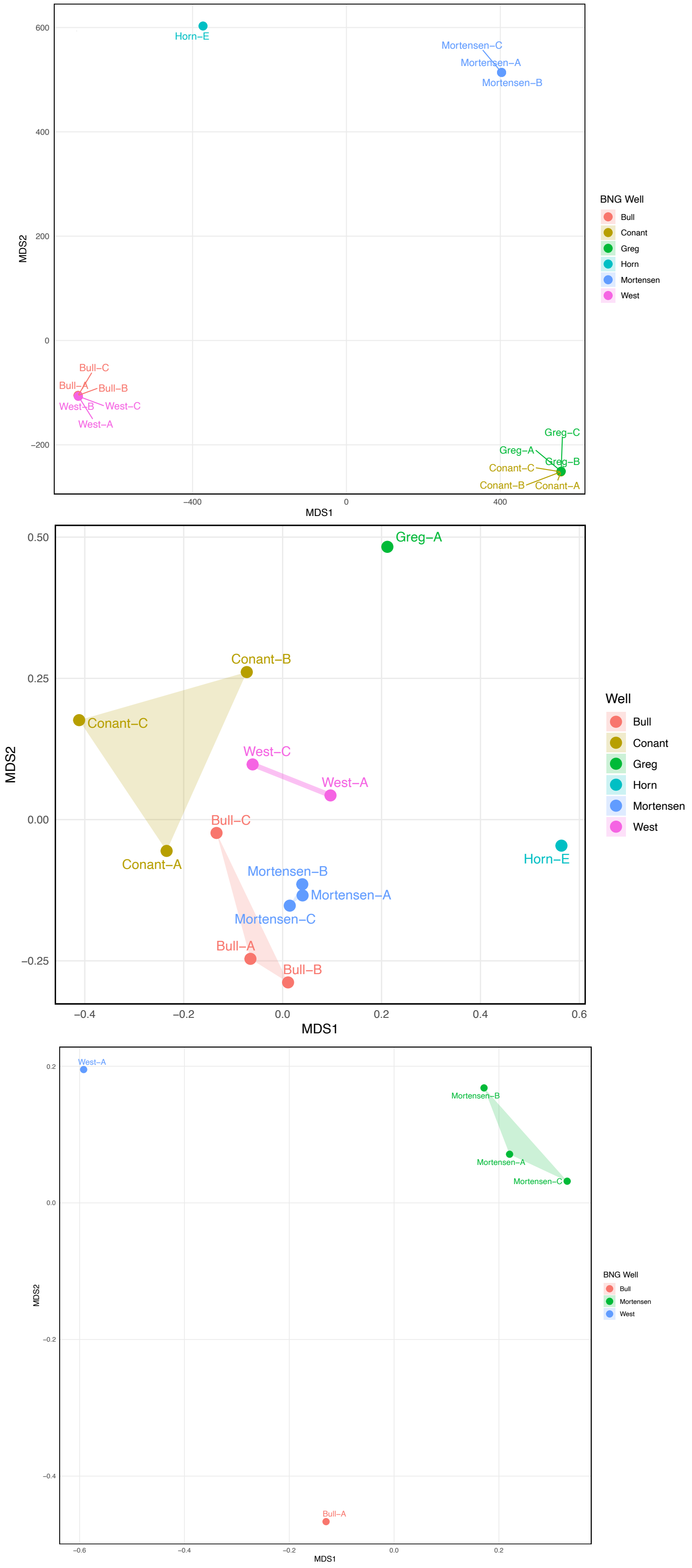

Supplementary Figure 4. Non-metric multidimensional scaling (NMDS) performed on 16S rRNA gene (A), ITS (B), and 18S rRNA gene (C) technical replicates that passed quality control, with replicates displaying strong clustering and subsequently pooled for downstream analyses.

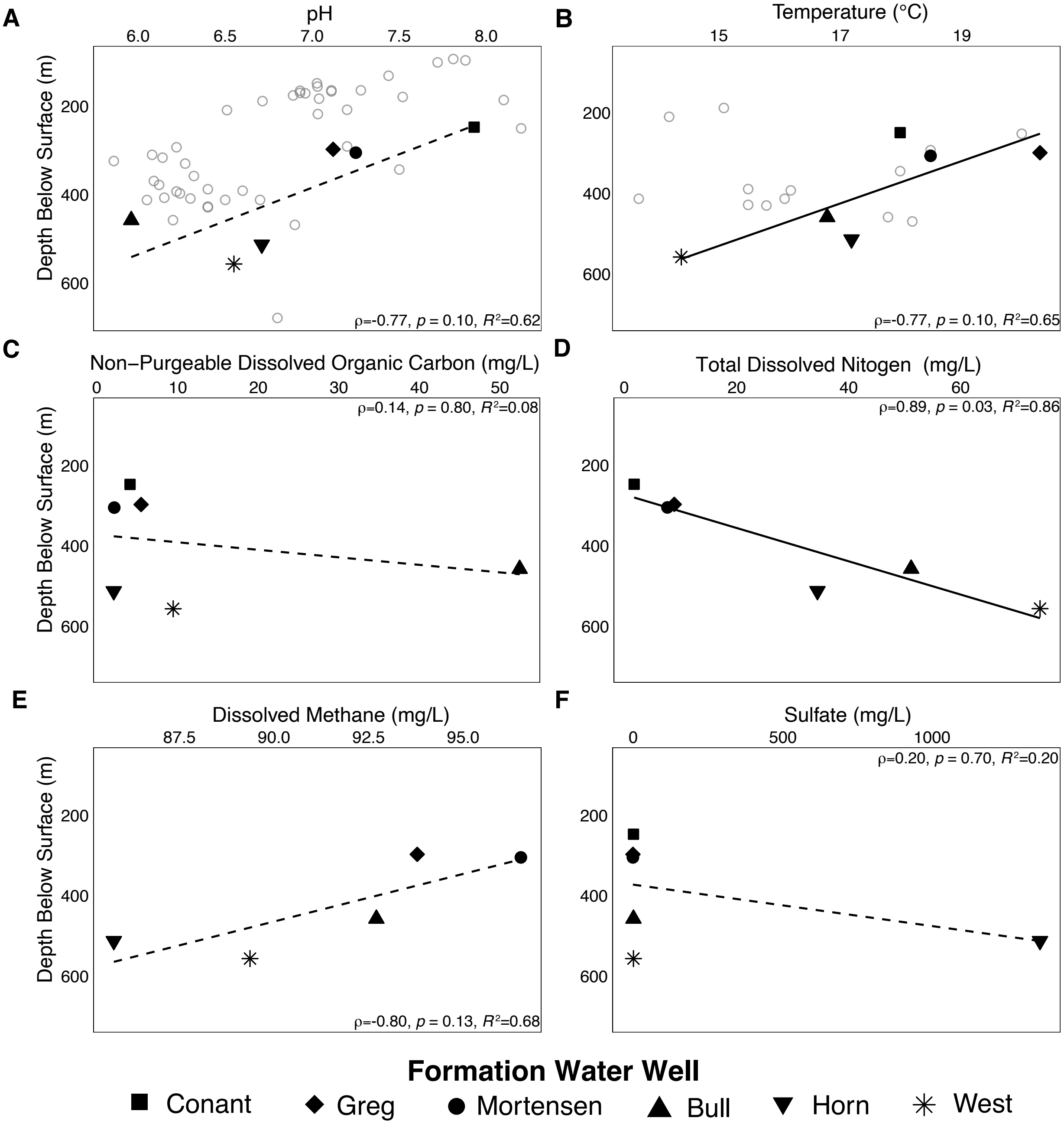

Supplementary Figure 5. Geochemical measures of formation water plotted against sample depth: (A) pH, (B) Temperature (°C), (C) Non-purgeable dissolved organic carbon (NPDOC), (D) Total dissolved nitrogen (TDN), (E) Dissolved methane, (F) Sulfate. For each variable, the Spearman correlation coefficient (ρ) and p-value for the association with well depth are indicated; significant correlations ($p < 0.05$) are shown with solid lines. The R^2 value from a linear regression fit is also reported. Black symbols represent new formation water data from this study, compared to previously published values for the Antrim Shale (open gray circles) [28–31].

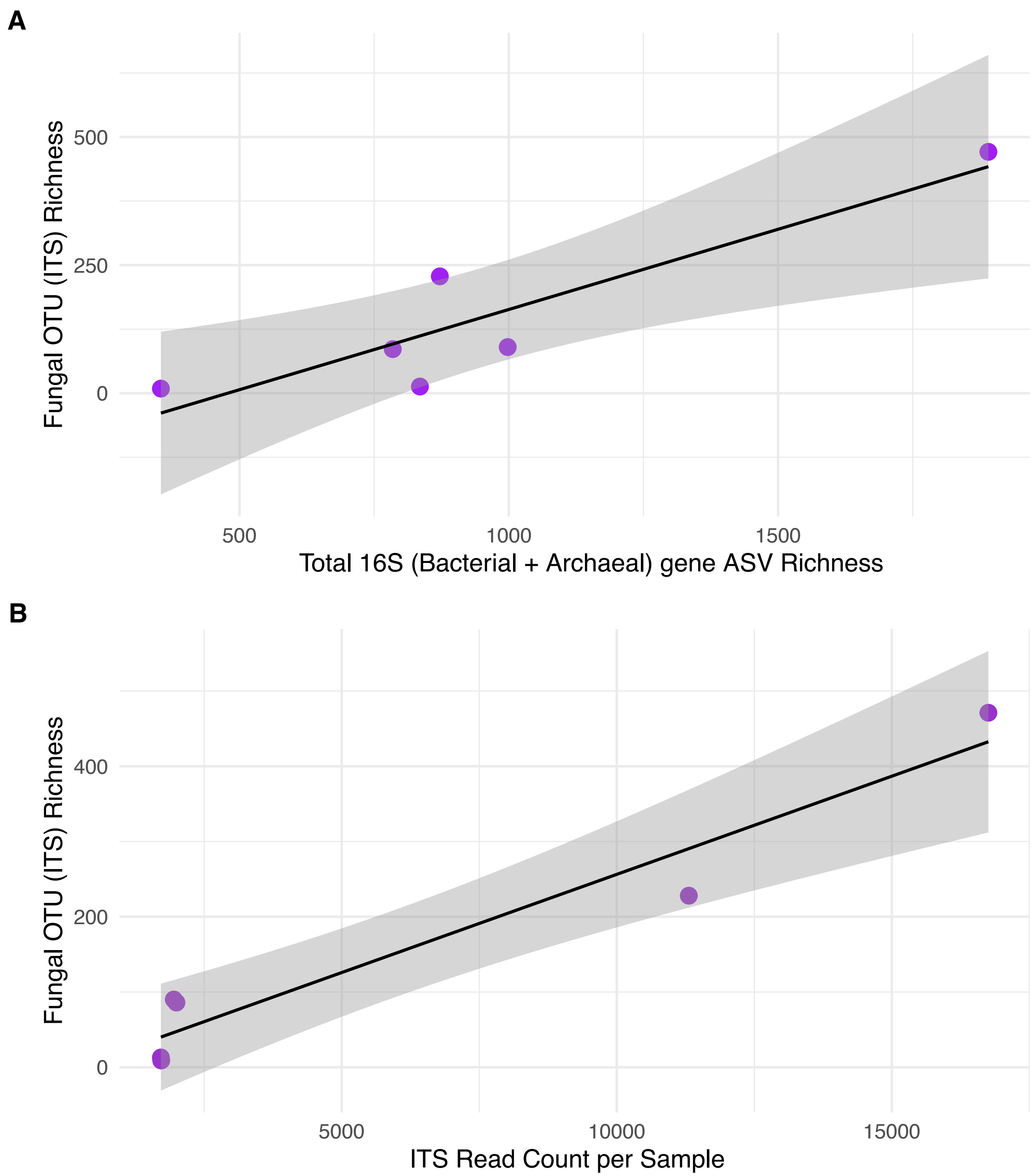

Supplementary Figure 6. (A) Relationship between 16S rRNA gene and ITS fungal richness across sampled formation water wells (p = 0.03, ρ = 0.89, R^2=0.81). (B) Relationship between ITS amplicon reads and observed fungal ITS richness per well (p = 0.03, ρ = 0.89, R^2=0.93). Shaded regions indicate the standard error of the linear regressions.

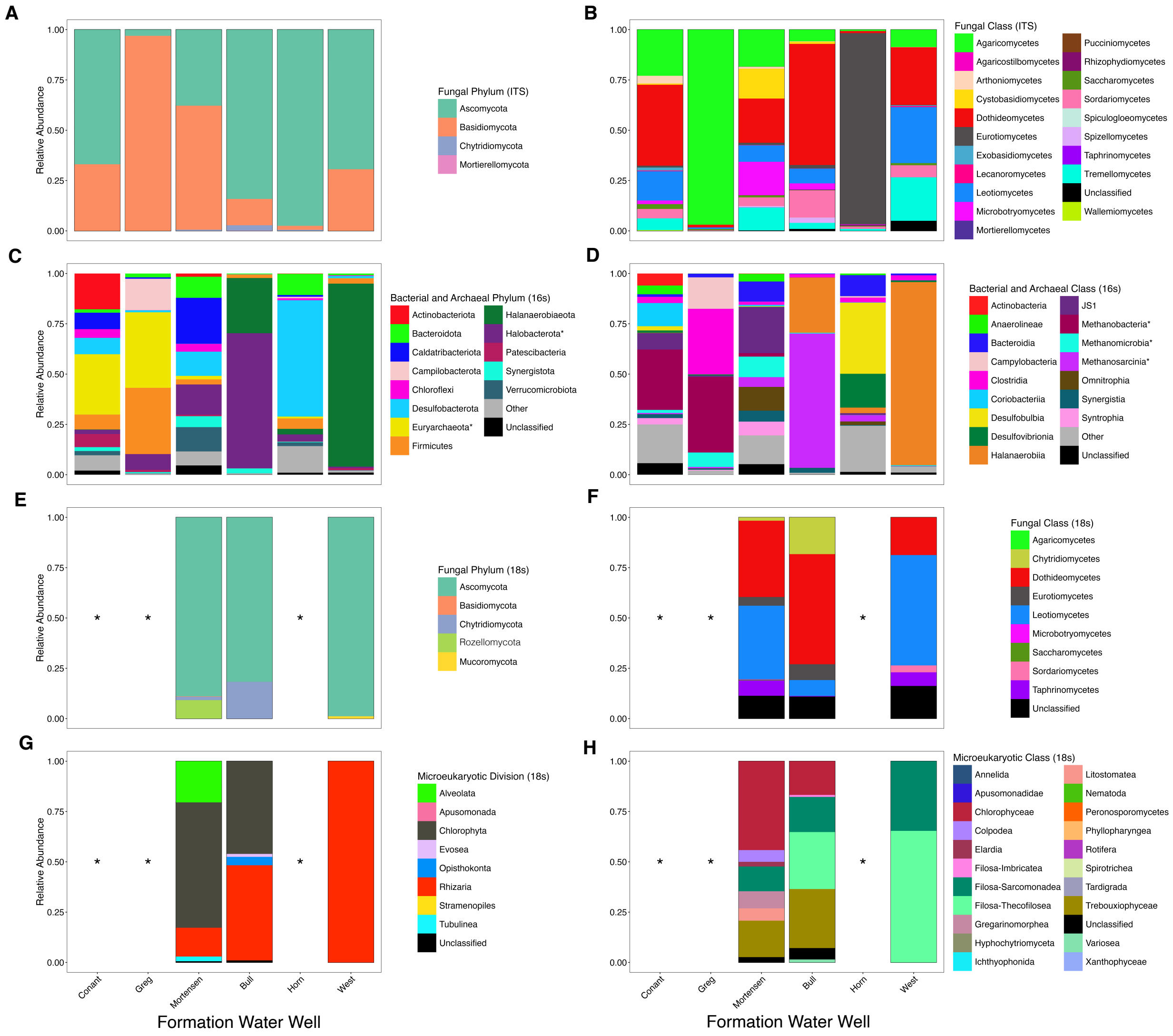

Supplementary Figure 7. Stacked bar plots displaying the relative abundances of microbial groups across sampled formation water wells. (A-B) Fungi profiled through ITS sequencing, (C-D) Bacteria and Archaea profiled through 16S rRNA gene sequencing, (E-F) Fungi and (G-H) Micro-eukaryotes profiled through 18S rRNA gene sequencing. Taxonomic groups comprising less than 1% of total reads are shown as "Other." Sites without any 18S technical replicates having sufficient reads (> 320 reads) are indicated with an asterisk (*).

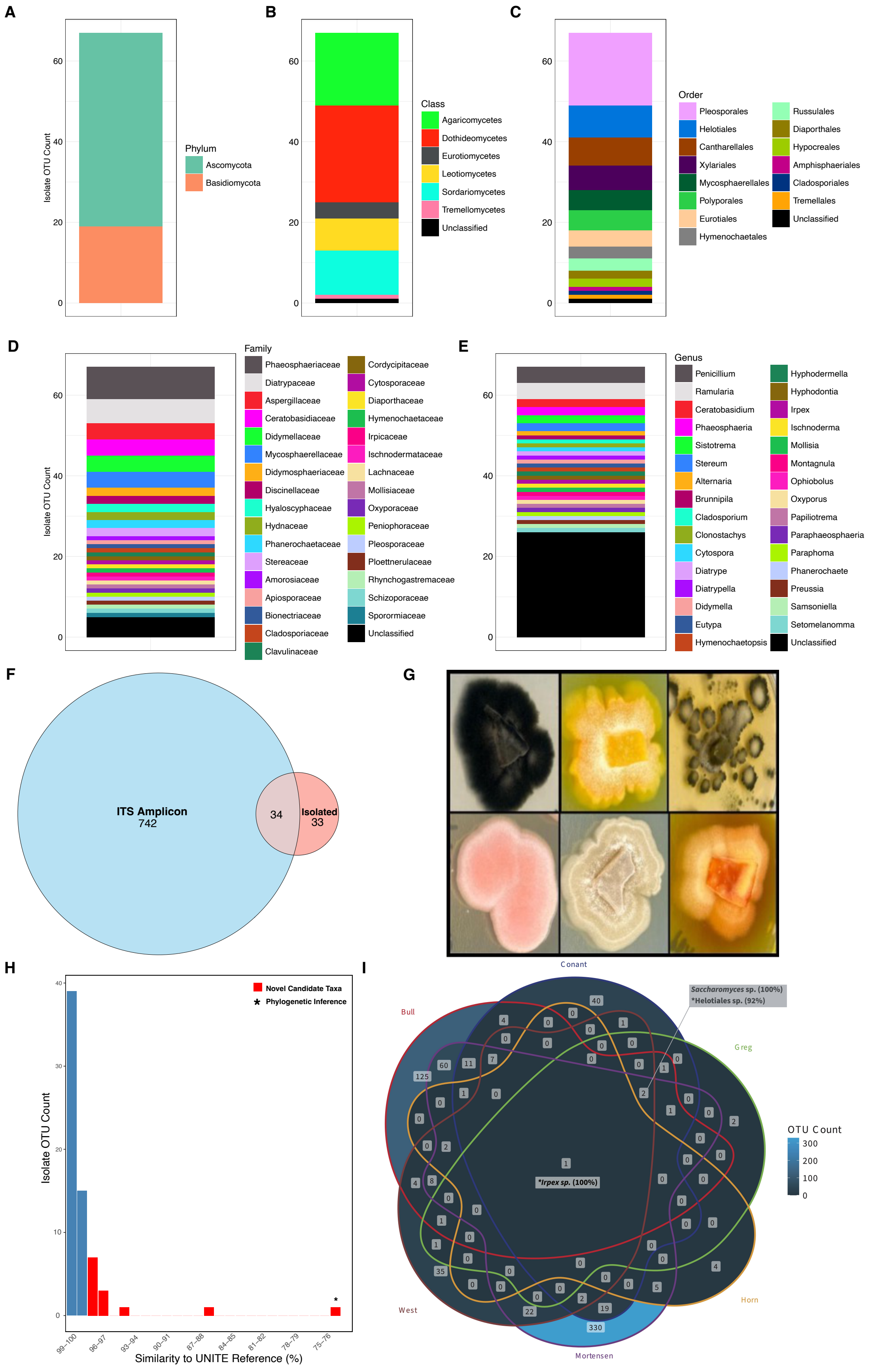
A
B
C
D
E
F
G
H
I
Isolate OTU Count
Phylum
Ascomycota
Basidiomycota
Class
Agaricomycetes
Dothideomycetes
Eurotiomycetes
Leotiomycetes
Sordariomycetes
Tremellomycetes
Unclassified
Order
Pleosporales
Helotiales
Cantharellales
Xylariales
Mycosphaerellales
Polyporales
Eurotiales
Hymenochaetales
Russulales
Diaporthales
Hypocreales
Amphisphaeriales
Cladosporiales
Tremellales
Unclassified
Family
Phaeosphaeriaceae
Diatrypaceae
Aspergillaceae
Ceratobasidiaceae
Didymellaceae
Mycosphaerellaceae
Didymosphaeriaceae
Discinellaceae
Hyaloscyphaceae
Hydnaceae
Phanerochaetaceae
Stereaceae
Amorosiaceae
Apiosporaceae
Bionectriaceae
Cladosporiaceae
Clavulinaceae
Cordycipitaceae
Cytosporaceae
Diaporthaceae
Hymenochaetaceae
Irpicaceae
Ischnodermataceae
Lachnaceae
Mollisiaceae
Oxyporaceae
Peniophoraceae
Pleosporaceae
Ploettnerulaceae
Rhynchogastremaceae
Schizoporaceae
Sporormiaceae
Unclassified
Genus
Penicillium
Ramularia
Ceratobasidium
Phaeosphaeria
Sistotrema
Stereum
Alternaria
Brunnipila
Cladosporium
Clonostachys
Cytospora
Diatrype
Diatrypella
Didymella
Eutypa
Hymenochaetopsis
Hyphodermella
Hyphodontia
Irpex
Ischnoderma
Mollisia
Montagnula
Ophiobolus
Oxyporus
Papiliotrema
Paraphaeosphaeria
Paraphoma
Phanerochaete
Preussia
Samsoniella
Setomelanomma
Unclassified
ITS Amplicon
742
34
Isolated
33
Novel Candidate Taxa
Phylogenetic Inference
Similarity to UNITE Reference (%)
99–100
96–97
93–94
90–91
87–88
84–85
81–82
78–79
75–76
Conant
Bull
Greg
Horn
West
Mortensen
Saccharomyces sp. (100%)
*Helotiales sp. (92%)
*Irpex sp. (100%)
OTU Count
300
200
100
0

Supplementary Figure 8. Multi-panel plot displaying characteristics of fungi isolated from sampled transect wells. (A-E) Taxonomic stacked bar plots of isolates summarized at various taxonomic levels. (F) Fraction of isolate OTUs that match amplicon OTUs. (G) Photos of representative fungal cultures. Photographs by the authors. (H) Distribution of sequence similarity between isolates and entries in the UNITE fungal database. (I) Number of amplicon OTUs shared among sites, with the single OTU found at all sites and the two OTUs present at five out of six sites highlighted; an asterisk (*) denotes that a representative of that OTU was successfully cultured.

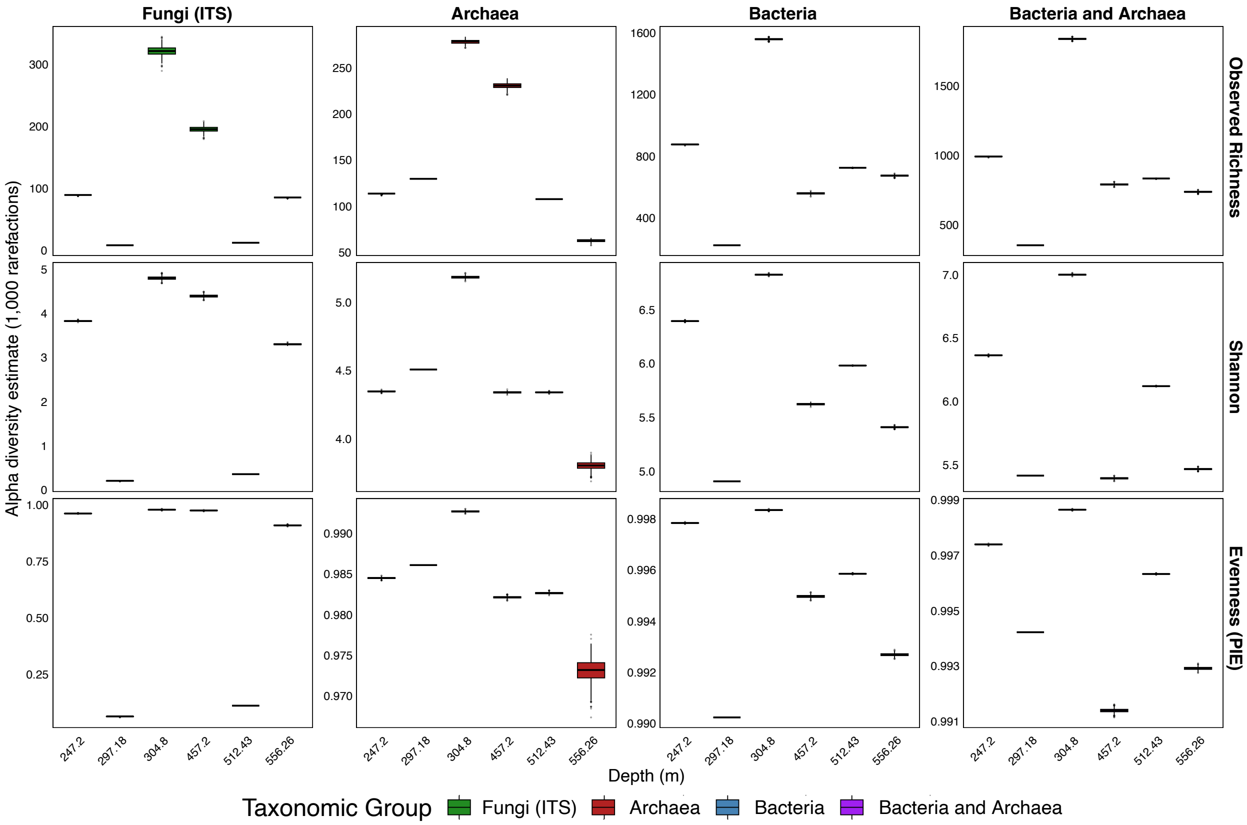

Supplementary Figure 9. Alpha-diversity estimates by analysis and taxonomic group. Alpha-diversity estimates are shown separately for each analysis and taxonomic group. Estimates were calculated from 1,000 repeated rarefactions; for each marker gene, samples were randomly subsampled to the minimum read depth observed for that group: 36,249 reads for 16S rRNA gene datasets and 1,690 reads for ITS datasets. Box-and-whisker plots display the distribution of estimates across rarefactions.

Supplementary Tables

Supplementary Table 1. Complete **aqueous** geochemistry and stable isotope values including total dissolved solids (TDS), non-purgeable dissolved organic carbon (NPDOC), dissolved inorganic carbon (DIC), alkalinity, total dissolved nitrogen (TDN).

Well Name	Conant D1-10	Gregory C3-12	Mortensen A4-24	Sitting Bull D4-4	Little Big Horn B4-23	Mancelona West D2-31
Field-ID	MI-04-060424	MI-05-060424	MI-02-060424	MI-06-060524	MI-07-060524	MI-08-060524
Depth (m)	247.2	297.18	304.8	457.2	512.43	556.26
TDS (mg/L)	1900	26300	20900	157000	88500	185000
pH	7.93	7.12	7.25	5.96	6.71	6.55
Latitude	45.0938	45.0105	44.9879	44.9344	44.8983	44.8598
Longitude	-85.2819	-85.2181	-85.2139	-85.1551	-85.1144	-85.083
Temperature (°C)	18	20.3	18.5	16.8	17.2	14.4
Bromide (mg/L)	0.015	25.1	12.3	211	140	356
Chloride (mg/L)	448	14600	11600	92100	50200	112000
DIC (mg C/L)	128.9	254	283.4	132.9	190.5	83.13
DIC (mmol/L)	10.7	21.1	23.6	11.1	15.9	6.9
Alkinity (mg CaCO3/L)	584.7	1190	1371	528.9	742.7	350
Alkinity (meq/L)	11.7	23.8	27.4	10.6	14.8	7
NPDOC (mg C/L)	4.184	5.558	2.224	52.56	2.163	9.54
Ammonium (mg/L)	0.639	5.467	6.924	53.88	31.39	0.915
SO_4^{2-} (mg/L)	2.08	0.3	0.2	2	1370	2
O_2 (%)	-	0.57	0.38	0.14	0.57	0.31
Methane (%)	-	93.83	96.57	92.75	85.81	89.41
TDN (mg N/L)	1.84	9.003	7.789	51.33	34.59	74.36
$\delta^{13}C\text{-}CH_4$	NA	-50.59	-51.09	-49.85	-50.99	-51.41
$\delta^{13}C\text{-}CO_2$	NA	23.07	22.4	23.22	21.75	21.1
$\delta^{18}O\text{-}H_2O$ (‰ VSMOW)	-14.55	-13.21	-12.25	-5.75	-10.38	-7.25
$\delta^{18}O\text{-}H_2O$ (‰) precision	0.1	0.1	0.1	0.1	0.1	0.1
$\delta^{2}H\text{-}H_2O$ (‰ VSMOW)	-102.14	-89.55	-71.99	-53.74	-65.25	-44.68
$\delta^{2}H\text{-}H_2O$ (‰) precision	1	1	1	1	1	1
Tapp (Apparent temperature °C)	NA	28.6	49	36.8	47.8	70.6
Tapp positive error	NA	-4.6	-10	-7.9	-13.4	-12.2
Tapp negative error	NA	4.7	10.7	8.4	14.7	13.2
Specific conductance (mS/cm)	2.3	40.1	33.2	177.7	114.1	198.2

Supplementary Table 2. All fungal isolate operational taxonomic units (OTUs) generated, including: representative strain, taxonomy, internal transcribed spacer (ITS) sequence percent match to UNITE Database, GenBank accession numbers, University of Michigan (MICH) herbarium voucher numbers. Bold indicates taxa used for phylogenetic placement and * indicates candidate novel taxa.

OTU ID	Rep. Strain(s)	Class	Lowest Taxonomy	% Unite (ITS)	ITS Genbank accession	U. Michigan Voucher(s)
**QM01***	**Horn_10**	**Dothideomycetes**	***Teichospora* sp.**	**74.194**	**PX589740**	**346816**
QM36*	Mort_48	Pezizomycetes	Discinellaceae sp.	86.85	PX589741	346817
QM09*	West_41	Agaricomycetes	Peniophoraceae sp.	94.184	PX589742	346818
QM081720*	West_49	Leotiomycetes	Hyaloscyphaceae sp.	96.313	PX589743	346819
QMb58c8c*	Bull_45, Bull_52	Sordariomycetes	*Eutypa* sp.	96.491	PX589744	346820, 346821
QM19*	Bull_49	Dothideomycetes	*Paraphaeosphaeria* sp.	96.739	PX589745	346822
QM32*	West_16	Pezizomycetes	Discinellaceae sp.	97.215	PX589746	346823
QM17*	Bull_51	Sordariomycetes	Diatrypaceae sp.	97.468	PX589747	346824
QM33*	Mort_22	Dothideomycetes	*Phaeosphaeria* sp.	97.493	PX589748	346825
QMd3a692*	Bull_04	Dothideomycetes	Didymellaceae sp.	97.61	PX589749	346826
QM28*	Mort_03	Agaricomycetes	Ceratobasidiaceae sp.	97.624	PX589750	346827
QM15*	Bull_06	Sordariomycetes	Diaporthaceae sp.	97.655	PX589751	346828
QM24*	West_11	Agaricomycetes	*Stereum* sp.	97.807	PX589752	346829
QMe94542	Mort_11	Sordariomycetes	*Ramularia* sp.	98.095	PX589754	346830
QM16	Shook_16	Leotiomycetes	Hyaloscyphaceae sp.	98.333	PX589755	346831
QM14	Horn_12	Sordariomycetes	Diatrypaceae sp.	98.603	PX589756	346832
QM34	Bull_26	Dothideomycetes	*Didymella* sp.	98.619	PX589757	346833
QM3cac07	Bull_50, Mort_55	Dothideomycetes	*Phaeosphaeria* sp.	98.624	PX589758	346834, 346835
QM22	Bull_22, Bull_25	Pleosporales	Didymellaceae sp.	98.649	PX589759	346836, 346896
QMbfc7d9	Bull_31	Agaricomycetes	*Oxyporus* cf. *corticola*	98.662	PX589760	346837
QM25	West_42	Leotiomycetes	*Mollisia* cf. *minutella*	98.664	PX589761	346838
QMf98e9d	Bull_17	Sordariomycetes	*Diatrype* sp.	98.673	PX589762	346839
QM7455ff	Bull_23, West_29	Sordariomycetes	*Clonostachys* sp.	98.729	PX589763	346840, 346841
QM08	Mort_37	Leotiomycetes	Ploettnerulaceae sp.	98.76	PX589764	346842
QM31	West_18	Eurotiomycetes	*Penicillium* sp.	98.765	PX589765	346843
QM35	Bull_19	Sordariomycetes	*Diatrypella* sp.	98.854	PX589766	346844
QM07	West_66	Agaricomycetes	Clavulinaceae sp.	98.947	PX589767	346845
QM04	Shook_13	Agaricomycetes	*Hymenochaetopsis* sp.	99.016	PX589768	346846
QM18936f	West_30	Dothideomycetes	*Paraphoma* sp.	99.052	PX589769	346847
QM47c26f	Mort_09	Dothideomycetes	Phaeosphaeriaceae sp.	99.091	PX589770	346848
QM157e2b	Bull_20	Sordariomycetes	*Apiospora* cf. *arundinis*	99.099	PX589771	346849
QM30	Mort_19	Eurotiomycetes	*Penicillium* cf. *bialowiezense*	99.111	PX589772	346850
QM21	Shooks_05	Dothideomycetes	*Cladosporium* sp.	99.142	PX589773	346851
QM4bb741	West_28	Agaricomycetes	*Stereum* cf. complicatum	99.286	PX589774	346852
QMa1a000	West_02	Agaricomycetes	*Ceratobasidium* sp.	99.306	PX589775	346853
QM11	Shook_08, Shook_1(	Eurotiomycetes	*Penicillium* sp.	99.399	PX589776	346854, 346855
QM06	West_09, West_01	Agaricomycetes	Ceratobasidiaceae sp.	99.431	PX589777	346856, 346857
QM02	Mort_33	Leotiomycetes	Helotiales sp.	99.443	PX589778	346858
QM157d09	Mort_30	Dothideomycetes	*Ramularia* cf. nyssicola	99.528	PX589779	346859
QM27	Mort_24	Dothideomycetes	*Ramularia* sp.	99.534	PX589780	346860
QM464a07	Shook_14	Dothideomycetes	Amorosiaceae sp.	99.563	PX589781	346861
QM98b09f	Bull_12	Eurotiomycetes	*Penicillium* sp.	99.567	PX589782	346862
QM18	Horn_13	Dothideomycetes	*Preussia* cf. *terricola*	99.574	PX589783	346863
QM02094e	Shook_07	Dothideomycetes	*Montagnula* sp.	99.587	PX589784	346864
QM934b36	Mort_31	Tremellomycetes	*Papiliotrema* sp.	99.595	PX589785	346865
QMfc8291	Bull_10	Dothideomycetes	*Leptosphaerulina* sp.	99.602	PX589786	346866
QM5fc928	West_06	Agaricomycetes	*Hyphodermella* cf. *rosae*	99.632	PX589787	346867
QMe19e53	Shook_12	Agaricomycetes	*Hyphodontia* sp.	99.64	PX589788	346868
QM05	Bull_07, Bull_15	Agaricomycetes	*Ceratobasidium* sp.	99.642	PX589789	346869, 346870
QM1aa54a	West_59	Agaricomycetes	*Sistotrema* sp.	99.65	PX589790	346871
QM018c2a	Shook_15	Dothideomycetes	Mycosphaerellales sp.	100	PX589791	346872
QM0fac24	West_08, Mort_16	Agaricomycetes	*Irpex* cf. lacteus	100	PX589792	346873, 346874
QM41f0cd	West_64	Dothideomycetes	*Ramularia* sp.	100	PX589793	346875
QM6b7bb4	Bull_08	Agaricomycetes	*Phanerochaete* cf. sordida	100	PX589794	346876
QM752a99	Mort_06	Agaricomycetes	*Gloeoporus* cf. *pannocinctus*	100	PX589795	346877
QM797347	Horn_05	Dothideomycetes	*Setomelanomma* cf. *holmii*	100	PX589796	346878
QM878f0a	West_24	Dothideomycetes	Phaeosphaeriaceae sp.	100	PX589797	346879
QMbd2852	West_60	Agaricomycetes	*Sistotrema* cf. *brinkmannii*	100	PX589798	346880
QMc73608	Mort_07	Agaricomycetes	*Ischnoderma* sp.	100	PX589799	346881
QMd034dc	Mort_04	Dothideomycetes	Phaeosphaeriaceae sp.	100	PX589800	346882
QMd86119	Bull_13	Sordariomycetes	*Cytospora* sp.	100	PX589801	346883
QMdafde3	Shook_04, Horn_01	Dothideomycetes	*Alternaria* sp.	100	PX589802	346884, 346885
QM10	Bull_14, Bull_30	Dothideomycetes	*Ophiobolus* sp.	100	PX589803	346886, 346887
QM13	Horn_11	Dothideomycetes	Pleosporales sp.	100	PX589804	346888
QM23	West_05	Sordariomycetes	*Samsoniella* sp.	100	PX589805	346889
QM29	Bull_46	Leotiomycetes	*Brunnipila* cf. *palearum*	100	PX589806	346890

Supplementary Table 3. Complete list of taxa used in phylogenetic placement of *Teichospora* sp. QM 01, with strain and large ribosomal subunit (LSU) NCBI Genbank accession numbers. Bold indicates sequences generated in this study.

Taxa names	Strain/voucher	LSU GenBank Accession
Teichospora sp.	**QM Horn_10**	**PX589807**
T. austroafricana	CBS 119330^T	EU552115
T. austroafricana	CBS 122674	EU552116
T. claviformis	GKM 1210^T	GU385212
T. grandicipis	CPC 1852	JN712520
T. grandicipis	CBS 111702^T	JN712521
T. kingiae	CPC 29104^T	KY173557
T. proteae	CBS 122675^T	EU552117
T. trabicola	C 141	KU601592
T. trabicola	C 157	KU601593
T. trabicola	CBS 140730^T	KU601591
Asymmetrispora mariae	CBS 140732^T	KU601580
Aurantiascoma minimum	GKM 169N^T	GU385165
Au. nephelii	CPC 27539^T	KY173558
Au. quercus	CBS 143396^T	MH107966
Decaisnella formosa	BCC 25617	GQ925847.1
Floricola striata	JK 5603K	GU479785
F. striata	JK 5678I	GU301813
Paulkirkia arundinis	MFLU 13-0315^T	KU848206.1
Magnibotryascoma kunmingense	KUMCC 20-0254^T	MW424784
M. acaciae	CPC 24801^T	KR611898
Misturatosphaeria radicans	ATCC 42522^T	U43479
Pseudoaurantiascoma kenyensis	GKM 1195^T	GU385194
Pseudomisturatosphaeria cruciformis	SMH 5151^T	GU385211
Ramusculicola thailandica	MFLUCC 13-0284^T	KP888647
Westerdykella cylindrica	CBS 454.72^T	NG_027595.1

References

1. Varonka MS et al. Geochemical Data for Produced Water and Gas from the Antrim Shale in Michigan, 2024. *US Geological Survey (USGS) Data Release.* 2026;158. https://doi.org/10.5066/P1AUEAYP
2. James TY et al. Evolutionary significance of imbalanced nuclear ratios in heterokaryons of Heterobasidion Parviporum. *Evol.* 2008;62:2279–2296. https://doi.org/10.1111/j.1558-5646.2008.00462.x
3. White TJ. Amplification and direct sequencing of fungal ribosomal RNA genes for phylogenetics. *PCR Protocols.* Academic Press, 1990, 315–322.
4. Kearse M, Moir R, Wilson A, Stones-Havas S, Cheung M, Sturrock S, et al. Geneious Basic: an integrated and extendable desktop software platform for the organization and analysis of sequence data. *Bioinformatics*. 2012;28:1647–1649.
5. Rodríguez-Ramos JA et al. Genome-Resolved Metaproteomics Decodes the Microbial and Viral Contributions to Coupled Carbon and Nitrogen Cycling in River Sediments. *mSystems.* 2022;7:e00516-22. https://doi.org/10.1128/msystems.00516-22
6. Op De Beeck M et al. Comparison and validation of some ITS primer pairs useful for fungal metabarcoding studies. *PLoS One.* 2014;9:e97629. https://doi.org/10.1371/journal.pone.0097629
7. Walters W et al. Improved Bacterial 16S rRNA Gene (V4 and V4-5) and Fungal Internal Transcribed Spacer Marker Gene Primers for Microbial Community Surveys. *mSystems.* 2015;1:10.1128/msystems.00009-15. https://doi.org/10.1128/msystems.00009-15
8. Comeau AM et al. Arctic Ocean Microbial Community Structure before and after the 2007 Record Sea Ice Minimum. *PLOS ONE.* 2011;6:e27492. https://doi.org/10.1371/journal.pone.0027492
9. Liu CM et al. BactQuant: An enhanced broad-coverage bacterial quantitative real-time PCR assay. *BMC Microbiol.* 2012;12:56. https://doi.org/10.1186/1471-2180-12-56
10. Liu CM et al. FungiQuant: A broad-coverage fungal quantitative real-time PCR assay. *BMC Microbiol.* 2012;12:255. https://doi.org/10.1186/1471-2180-12-255
11. Lofgren LA et al. Genome-based estimates of fungal rDNA copy number variation across phylogenetic scales and ecological lifestyles. *Mol Ecol.* 2019;28:721–730. https://doi.org/10.1111/mec.14995
12. Stoddard SF et al. rrnDB: improved tools for interpreting rRNA gene abundance in bacteria and archaea and a new foundation for future development. *Nucleic Acids Res.* 2015;43:D593–D598. https://doi.org/10.1093/nar/gku1201
13. Breyer E et al. The contribution of pelagic fungi to ocean biomass. *Cell.* 2025;188:3992-4002.e13. https://doi.org/10.1016/j.cell.2025.05.004
14. Ducklow H. Bacterial Production and Biomass in the Oceans. *Microbial Ecology of the Oceans, DL Kirchman, ed* 2000;85–120.
15. Bolyen E et al. Reproducible, interactive, scalable and extensible microbiome data science using QIIME 2. *Nat Biotechnol.* 2019;37:852–857. https://doi.org/10.1038/s41587-019-0209-9
16. Rognes T et al. VSEARCH: a versatile open source tool for metagenomics. *PeerJ.* 2016;4:e2584. https://doi.org/10.7717/peerj.2584
17. Tedersoo L et al. Global patterns in endemicity and vulnerability of soil fungi. *Glo Change Biol.* 2022;28:6696–6710. https://doi.org/10.1111/gcb.16398

18. Tedersoo L et al. Best practices in metabarcoding of fungi: From experimental design to results. *Mol Ecol.* 2022;31:2769–2795. https://doi.org/10.1111/mec.16460
19. Quast C et al. The SILVA ribosomal RNA gene database project: improved data processing and web-based tools. *Nucleic Acids Res.* 2013;41:D590–D596. https://doi.org/10.1093/nar/gks1219
20. Guillou L et al. The Protist Ribosomal Reference database (PR2): a catalog of unicellular eukaryote Small Sub-Unit rRNA sequences with curated taxonomy. *Nucleic Acids Res.* 2013;41:D597–D604. https://doi.org/10.1093/nar/gks1160
21. Abarenkov K et al. The UNITE database for molecular identification and taxonomic communication of fungi and other eukaryotes: sequences, taxa and classifications reconsidered. *Nucleic Acids Res.* 2024;52:D791–D797. https://doi.org/10.1093/nar/gkad1039
22. Tedersoo L et al. The Global Soil Mycobiome consortium dataset for boosting fungal diversity research. *Fungal Divers.* 2021;111:573–588. https://doi.org/10.1007/s13225-021-00493-7
23. R Core Team. R: A language and environment for statistical computing. R Foundation for Statistical Computing, Vienna, Austria. https://www.R-project.org/ 2024.
24. Dixon P. VEGAN, A Package of R Functions for Community Ecology. *J Veg Sci.* 2003;14:927–930. https://doi.org/10.1111/j.1654-1103.2003.tb02228.x
25. Wickham H. Data Analysis. In: Wickham H (ed.), ggplot2: Elegant Graphics for Data Analysis. Cham: Springer International Publishing, 2016, 189–201.
26. McMurdie PJ, Holmes S. phyloseq: An R Package for Reproducible Interactive Analysis and Graphics of Microbiome Census Data. *PLoS ONE.* 2013;8:e61217. https://doi.org/10.1371/journal.pone.0061217
27. Foster ZSL, Sharpton TJ, Grünwald NJ. Metacoder: An R package for visualization and manipulation of community taxonomic diversity data. *PLOS Comput Biol.* 2017;13:e1005404. https://doi.org/10.1371/journal.pcbi.1005404
28. Martini AM et al. Microbial production and modification of gases in sedimentary basins: A geochemical case study from a Devonian shale gas play, Michigan basin. *AAPG Bull.* 2003;87:1355–1375. https://doi.org/10.1306/031903200184
29. Martini AM, Walter LM, McIntosh JC. Identification of microbial and thermogenic gas components from Upper Devonian black shale cores, Illinois and Michigan basins. *AAPG Bull.* 2008;92:327–339. https://doi.org/10.1306/10180706037
30. McIntosh JC, Garven G, Hanor JS. Impacts of Pleistocene glaciation on large-scale groundwater flow and salinity in the Michigan Basin. *Geofluids* 2011;11:18–33. https://doi.org/10.1111/j.1468-8123.2010.00303.x
31. McIntosh JC, Walter LM, Martini AM. Extensive microbial modification of formation water geochemistry: Case study from a Midcontinent sedimentary basin, United States. *GSA Bull.* 2004;116:743–759. https://doi.org/10.1130/B25371.1
